# Multivariate Analysis, Mass Balance Techniques, and Statistical Tests as Tools in Igneous Petrology: Application to the Sierra de las Cruces Volcanic Range (Mexican Volcanic Belt)

**DOI:** 10.1155/2014/793236

**Published:** 2014-03-05

**Authors:** Fernando Velasco-Tapia

**Affiliations:** Universidad Autónoma de Nuevo León, Facultad de Ciencias de la Tierra, Ex-Hacienda de Guadalupe, Carretera Linares-Cerro Prieto km 8, 67700 Linares, NL, Mexico

## Abstract

Magmatic processes have usually been identified and evaluated using qualitative or semiquantitative geochemical or isotopic tools based on a restricted number of variables. However, a more complete and quantitative view could be reached applying multivariate analysis, mass balance techniques, and statistical tests. As an example, in this work a statistical and quantitative scheme is applied to analyze the geochemical features for the Sierra de las Cruces (SC) volcanic range (Mexican Volcanic Belt). In this locality, the volcanic activity (3.7 to 0.5 Ma) was dominantly dacitic, but the presence of spheroidal andesitic enclaves and/or diverse disequilibrium features in majority of lavas confirms the operation of magma mixing/mingling. New discriminant-function-based multidimensional diagrams were used to discriminate tectonic setting. Statistical tests of discordancy and significance were applied to evaluate the influence of the subducting Cocos plate, which seems to be rather negligible for the SC magmas in relation to several major and trace elements. A cluster analysis following Ward's linkage rule was carried out to classify the SC volcanic rocks geochemical groups. Finally, two mass-balance schemes were applied for the quantitative evaluation of the proportion of the end-member components (dacitic and andesitic magmas) in the comingled lavas (binary mixtures).

## 1. Introduction

Several conventional mineralogical, geochemical, and isotopic tools, using a limited number of variables (e.g., bivariate, trilinear, multielement, and semilogarithmic diagrams), have usually been applied to establish a qualitative or semiquantitative view of igneous petrological mechanisms [1, 2]. Particularly, the interaction between, at least, two magmas is one of the most important mechanisms of compositional diversification of igneous rocks [3]. According to genetic relations between the original or *resident* magma and the later *invasive* magma, two scenarios could be expected [4, 5]: (a) successive pulses of magma derived from a common source intersect in time and space or (b) unrelated chemical distinct magmas, derived from different sources are involved in the interaction episode. Additionally, different styles of the interaction phenomena are related to the variation of physicochemical parameters (e.g., [3, 6, 7]): (a) the initial contrast in chemical composition, temperature, and viscosity, (b) the relative mass fractions and the physical state of interacting magmas, and (c) the static versus dynamic environment of interaction. These processes have been broadly divided into (a) *magma mingling*, a route characterized by a physical juxtaposition and intermingling of contrasting compositions, with little or no chemical homogenization, and (b) *magma mixing*, where the physical and chemical conditions promote the homogenization of contrasting geochemical and isotopic features, resulting in a single magma of intermediate composition. If a magma mixing/mingling model is proposed, it must include statements specifying (a) the initial compositions of the resident and invasive magmas, (b) the modal mineralogy of the magmas prior to mixing, and (c) the proportions of resident and invasive magmas [4]. A quantitative assessment could be obtained from multivariate statistical techniques [8]. Although these methods have been used with classification purposes in igneous rocks [9], their use to understand magma mixing/mingling processes is still limited [7, 10–13].

On the other hand, magma mixing/mingling processes have been observed in diverse tectonic settings. Consequently, a complete vision of these magmatic localities, commonly dominated by rocks with [SiO_2_]_adj_ > 52% (the subscript _adj_ refers to the adjusted silica from the SINCLAS computer program [14, 15]), would be facilitated from the tectonic regime. However, a restricted number of conventional diagrams are available for tectonic discrimination of intermediate ([SiO_2_]_adj_ = 52–63%; [16, 17]) and acid ([SiO_2_]_adj_ > 63%; [1, 18]) magmas. Additionally, these schemes have been critiqued as a result of a statistically wrong treatment of compositional data, eye-drawn subjective boundaries for different tectonic fields, and lack of representation of the entire statistical population [19, 20]. S. P. Verma and S. K. Verma [21] and Verma et al. [22], to solve the limitations of the tectonic discrimination conventional schemes, have proposed a set of new discriminant-function-based multidimensional diagrams for intermediate and acid magmas from four tectonic settings (island arc, continental arc, continental rift + ocean island, and collision).

**Figure 1:**
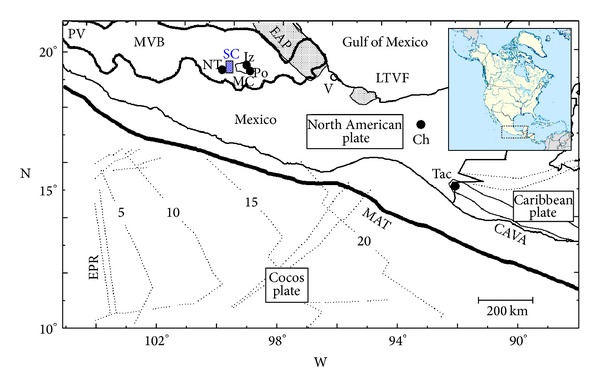
Location of the Sierra de las Cruces (SC) volcanic range (blue shaded box) at the central part of the Mexican Volcanic Belt (MVB) (modified from [30]). For guidance, the black box at the upper right side shows the location of this zone in North America. The figure also includes the approximate location of the Eastern Alkaline Province (EAP), Los Tuxtlas Volcanic Field (LTVF), Central American Volcanic Arc (CAVA), and the Chichón (Ch) and Tacaná (T) volcanoes. Other tectonic features are the Middle America Trench (MAT, shown by a thick black curve) and the East Pacific Rise (EPR, shown by a pair of dashed-dotted black lines). The traces marked by numbers 5 to 20 on the oceanic Cocos plate give the approximate age of the oceanic plate in Ma. Locations of Iztaccíhuatl (Iz), Popocatépetl (Po), and Nevado de Toluca (NT) are also shown. Cities are PV: Puerto Vallarta, MC: Mexico City, and V: Veracruz.

In this context, Velasco-Tapia et al. [23] recently reported, based on mineralogical, geochemical, and Sr-Nd isotopic conventional tools, that the formation of the Sierra de las Cruces (SC) volcanic range (3.7 to 0.5 Ma; central part of the Mexican Volcanic Belt (MVB); Figure 1) was mainly controlled by a magma mixing/mingling process. In this work, as an example, multivariate techniques (linear discriminant, cluster, and principal component analysis), discordancy and significance statistical tests, and mass-balance approaches were applied to establish the tectonic setting and to obtain a quantitative picture of the magmatic evolution of this volcanic range.

## 2. Geological Synthesis

**Figure 2:**
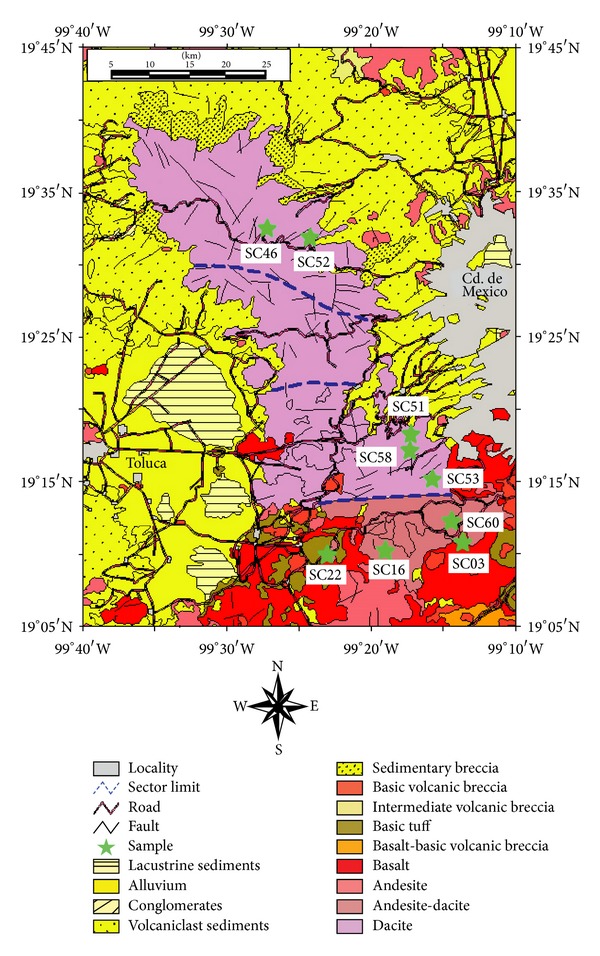
Geologic sketch of the Sierra de las Cruces volcanic range, showing lithology, faults, roads, and distribution of the samples (green stars) collected along the volcanic range in this work (modified from [23]). Study area division in four sectors from N to S based on K-Ar radiometric data [26]: (a) SCN-northern sector (2.9–3.7 Ma), (b) SCC-central sector (1.9–2.9 Ma), (c) SCS-southern sector (0.7–1.9 Ma), and (d) SCT-transition sector that include the Ajusco volcano (<0.7 Ma).

The SC volcanic range is an elongated volcanic range, extending in a NNW-SSE direction for ~65 km, with a width varying between 47 km to the north and 27 km to the south (Figure 2; [23–25]). According to K-Ar geochronological data [26], the main mass of SC volcanic range was erupted between 3.7 and 1.8 Ma. After that, in the middle Pleistocene (~0.5 Ma), another volcanic event produced andesitic domes, being labeled as Ajusco period. It has been considered as the transition to the Sierra de Chichinautzin monogenetic eruptive period (<40 ka; [27–29]).

On the basis of morphostructural and radiometric age criteria, the SC volcanic range has been divided into four sectors bounded by E-W faults [23, 24]: (a) northern sector (SCN; 2.9–3.7 Ma), (b) central sector (SCC; 1.9–2.9 Ma), (c) southern sector (SCS; 0.7–1.9 Ma), and (d) las Cruces-Chichinautzin transition sector (SCT; ~0.5 Ma). The northern and central sectors are characterized by morphostructures controlled by N-S and NE-SW fault systems. In contrast, E-W faults have ruled the morpholineaments and drainage patterns observed in the southern sector and the transition region.

The SC stratovolcanoes underwent alternated episodes, associated with faulting, of effusive and explosive activity. Porphyritic andesite to dacite lava flows (*Lava Dacítica Apilulco*; thickness < 4 m) with planar fracturing subparallel to the surface constitute the main effusive products. They generally show a mineralogical assemblage of plagioclase + amphibole + orthopyroxene ± clinopyroxene ± quartz + Fe-Ti oxides. Spherical to ellipsoidal magmatic enclaves occasionally occur in these lava flows. They are randomly distributed along the volcanic range, although the number and size apparently increase towards the north. Majority of the magmatic enclaves display a few millimeters to 4 centimeters in diameter, although in some northern outcrops they reach ~20 cm in diameter. The explosive products consist in pyroclastic deposits (*Brecha Piroclástica Cantimplora*; thickness = 1–4 m), conformed by dacitic blocks (20–30 cm), pumice clasts (<15 cm), and ash, that occurred intercalated with the lava flows.

Velasco-Tapia et al. [23] developed an extensive study in the SC volcanic range that includes detailed petrography, mineral chemistry, whole-rock geochemistry, and Sr-Nd isotopic data. These authors reported that several disequilibrium features confirm the significant role of the magma mingling/mixing processes between andesitic and dacitic magmas with concomitant fractional crystallization. The SC magmas were probably generated at different levels of the continental crust by partial melting. The magma mixing/mingling evidence includes (a) normal and sieved plagioclases in the same sample, rounded and embayed crystals, and armored rims over the dissolved crystal surfaces; (b) subrounded, vesicular magmatic enclaves, ranging from a few millimeters to ~20 centimeters in size (mineralogical assemblage: plagioclase + orthopyroxene + amphibole + quartz ± olivine ± Fe-Ti-oxides); (c) crystals with reaction rims or heterogeneous plagioclase compositions (inverse and oscillatory zoning or normally and inversely zoned crystals) in the same sample; and (d) elemental geochemical variations and trace element ratio more akin to magma mixing and to some extent diffusion process. Andesitic enclaves have been interpreted as portions of the intermediate magma that did not mix completely (mingling) with the felsic host lavas.

## 3. Methods

In the present work ten samples, collected along the SC volcanic range (Figure 2; SCN: SC46, SC52, and SC52a; SCS: SC51, SC53, and SC58; SCT: SC03, SC16, SC22, and SC60), were studied to obtain new petrographic and geochemical data. Modal compositions were determined by point counting on thin sections using a Prior Scientific petrographic microscope. Approximately 500 points per sample were counted in order to obtain a representative mode (Table 1).

Major and trace element composition of these SC volcanic rocks (Tables 2 and 3) were determined in ActLabs laboratories (Ancaster, Canada), using the “4LithoRes” methodology (for details consult webpage http://www.actlabsint.com/). Major elements were analyzed by inductively coupled plasma-optical emission spectrometry (ICP-OES) with an analytical precision <2% and accuracy typically better than 5% at 95% confidence level, based on analysis of diverse geochemical reference materials (GRM). Trace element concentrations were determined by inductively coupled plasma-mass spectrometry (ICP-MS) with an analytical precision 3–6% (occasionally reaching 9-10%) and an accuracy typically better than 7–12% for most elements at the 95% confidence level, based on analysis of diverse GRM.

## 4. Sierra de las Cruces Database and Evaluation Scheme

### 4.1. Mineralogical and Geochemical Database

A more complete SC database of the mineralogical modes and the whole-rock geochemical composition was established from the new as well as the published information reported by Velasco-Tapia et al. [23]. CIPW norms for samples were calculated on a 100% anhydrous adjusted basis of major element composition, with [Fe_2_O_3_]_adj_/[FeO]_adj_ ratios adjusted depending on the rock type [34]. Rock classification was based on the total alkali-silica (TAS) scheme [35, 36]. All computations (anhydrous and iron-oxidation ratio adjustments, norm compositions, and rock classifications) were automatically done using the SINCLAS software [14, 15].

### 4.2. Linear Discrimination Analysis

The tectonic affinity of the SC volcanic rocks was established applying new discriminant-function-based multidimensional diagrams for intermediate ([SiO_2_]_adj_ = 52–63%) and acid ([SiO_2_]_adj_ > 63%) rocks using the linear discriminant analysis (LDA) of natural logarithm ratios of major elements, immobile major and trace elements and immobile trace elements. These diagrams [21, 22] were proposed to discriminate island arc (IA), continental arc (CA), within-plate (continental rift, CR, and ocean island, OI, together), and collisional (Col) settings. Based on the earlier work of Verma and Agrawal [39] and the modifications outlined by Verma [40], these diagrams also provide probability estimates for individual samples, which were used in the present work.

Firstly, the nature of intermediate or acid magma for each sample was confirmed from the SINCLAS software [14, 15], under the Middlemost [34] option for Fe-oxidation adjustment. After that, a series of natural logarithms of element ratios were estimated for all samples. This transformation provided a Gaussian character to the distribution data, a basic condition of the LDA. After that, the ln-ratio data were used to estimate two discriminant functions (DF1 and DF2), obtained from the LDA (canonical analysis), and the individual probability for each sample to a tectonic regime. This statistical exercise was first performed to discriminate between IA + CA, CR + IO, and Col settings and four times for all possible combinations of three groups at a time out of four groups (IA, CA, CR + OI, and Col). Details of the statistical methodology and LDA equations have been reported in [21, 22]. It is important to note that the discrimination analysis was carried out considering the four SC sectors. All LDA equations were incorporated in a STATISTICA for Windows (Statsoft, Inc., Tulsa, OK, USA) spreadsheet and discrimination diagrams were constructed from these results.

### 4.3. Discordancy and Significance Tests

In order to better understand the contribution of the subducted Cocos plate to the SC magmas, the methodology put forth and practiced by Verma [38] was applied. This approach basically consists of comparing the magmas closer to the Middle America Trench (MAT) to those farther from it; that is, the SC sectors were statistically compared as two groups. The null hypothesis (H_0_: the two groups did not differ significantly at strict 99% confidence level) and the alternate hypothesis (H_A_: the two groups differ significantly at 99% confidence level) were tested by Fisher *F* and Student's *t*-tests (UDASYS software, [37]). Because the significance tests require that the data be normally distributed, single-outlier type discordancy tests were applied at strict 99% confidence level, for which DODESSYS software of Verma and Díaz-González [41] was used.

### 4.4. Cluster Analysis

The principal aim of this statistical tool is to partition observations into a number of groups. It is expected that the observations within a cluster are as similar as possible, whereas the differences between the clusters are as large as possible. In magma mingling scenario, this technique would be helpful for the SC sample distribution in resident, invasive, and comingled magmas.

In this work, cluster analysis was performed using the natural logarithm of major elements ([SiO_2_]_adj_–[P_2_O_5_]_adj_) and representative trace (transition: Co, V; rare earth: La, Eu, Yb; lithophile: Ba, Sr, U; high-field strength: Hf, Y, Zr) elements to [Al_2_O_3_]_adj_ ratios by using a hierarchical cluster method (HCM; [42]). Geochemical ratios were previously standardized (z-scores) by means of

(1) $$\begin{array}{c} K_{ij}=\frac{X_{ij}-X}{S_{ic}}, \end{array}$$

where *K*_*ij*_ is the standardized value of *X*_*ij*_, the *i*th variable for the *j*th sample, *X* is the mean value of the *i*th variable, and *S*_*ic*_ is its standard deviation. Additionally, the normality of each standardized variable was confirmed by the Shapiro-Wilks test. Cluster analysis applied a Ward's linkage rule, which linked iteratively nearby points through a similarity matrix and performed an ANOVA test to evaluate the distance between clusters [43]. The adopted procedure gives equal weight to each geochemical ratio. The measure of similarity was simply the distance as defined in Euclidean space. The distance between two samples (*j*, *k*) is given by

(2) $$\begin{array}{c} d_{jk}={\left[\sum_{i=1}^{N}{\left(K_{ij}-K_{ik}\right)}^{2}\right]}^{1/2}, \end{array}$$

where *K*_*ij*_ denotes the *K*th variable measured on object *i* in sample *j* and *K*_*jk*_ is the *K*th variable measured on object *i* in sample *k*. The results of the cluster analysis were graphically displayed in three dendrograms with units in Euclidean values, corresponding to northern, central, and southern-transition SC sectors.

The weight of geochemical log-ratios in the cluster approach was determined from the results obtained in a principal component analysis (PCA). It has been defined as an orthogonal linear transformation for reducing the dimensionality of a dataset by expressing it as the combination of a small number of linearly independent factors or “principal components.” Each factor will be a function of the individual contributions of the original variables [44]. The greatest variance for the transformed data was linked to the first principal component, whereas the second variance magnitude was related to the second principal component, and so on. The PCA considers a data matrix, *𝕏* (*n* rows × *p* columns; rows represent different samples, and columns give a particular chemical component; each component which has been standardized yielded a zero empirical mean). The transformation is stated by a set of *p*-dimensional vectors *𝕨*_(*k*)_ = (*w*_1_,…,*w*_*p*_)_(*k*)_ that map each row vector *𝕩*_(*i*)_ of *𝕏* to a new vector of principal component factors *𝕥*_(*i*)_ = (*t*_1_,…,*t*_*p*_)_(*i*)_ given by

(3) $$\begin{array}{c} {𝕥}_{k(i)}={𝕩}_{\left(i\right)}\cdot {𝕨}_{\left(k\right)}. \end{array}$$

Individual variables of *𝕥* considered over the data set successively inherit the maximum possible variance from *𝕩*, with each loading *𝕨* constrained to be a unit vector. The first principal component *𝕨*_(1)_ satisfied

(4) $$\begin{array}{c} {𝕨}_{\left(1\right)}=\arg\max\left\{\sum_{i}{\left(t_{1}\right)}_{\left(i\right)}^{2}\right\}=\arg\max\sum_{i}{\left({𝕩}_{\left(i\right)}\cdot 𝕨\right)}^{2}, \end{array}$$

where the quantity to be maximized is known as Rayleigh quotient. The *k*th component was determined by subtracting the *k* – 1 principal components from *𝕏*:

(5) $$\begin{array}{c} {\hat{𝕏}}_{k-1}=𝕏-\sum_{s=1}^{k-1}𝕏{𝕨}_{\left(s\right)}{𝕨}_{\left(s\right)}^{𝕋}. \end{array}$$

The vector associated with this component and showing the maximum variance from this new matrix would be defined as

(6) $$\begin{array}{c} {𝕨}_{\left(k\right)}=\arg\max\left\{{||{\hat{𝕏}}_{k-1}𝕨||}^{2}\right\}. \end{array}$$

All calculations related to cluster analysis were carried out using the STATISTICA for Windows software.

### 4.5. Mass-Balance Evaluations

Nixon [31] applied a simple mass-balance scheme for the quantitative characterization of binary mixtures and end-member compositions in the Iztaccíhuatl volcano (central MVB). The author suggested that, despite the compositional heterogeneity, if a chemical component can be found whose concentration is invariant in time and known in the mix and in each of the end-members, it is possible to treat quantitatively the magma mixing process.

Mixing proportions may be calculated considering the lever principle and the composition of the comingled magma subsequently described for all chemical components. The amount of a component in the mixed lava could be represented by

(7) $$\begin{array}{c} Q_{A}^{i}=\frac{\left|C_{M}^{i}-C_{B}^{i}\right|}{\left|C_{A}^{i}-C_{B}^{i}\right|}, \end{array}$$

where *Q*_*A*_^*i*^ + *Q*_*B*_^*i*^ = 1, and *Q* ^*i*^ and *C* ^*i*^ represent the weight fraction and concentration, respectively, of element *i* in subscripted end-members *A* and *B* and mixture *M*. The composition of an end-member could be estimated by

(8) $$\begin{array}{c} C_{A}^{j}=\frac{\left|C_{M}^{j}-Q_{B}^{i}C_{B}^{j}\right|}{Q_{A}^{i}}, \end{array}$$

where constituent *i* ≠ *j*. In this work, this mass-balance approach (model A) was applied to SC lavas, being restricted to those sectors where the end-member compositions were available and to those components that exhibit a statistically significant linear coherence in [SiO_2_]_adj_-Harker diagrams. This test involved the evaluation, at 99% confidence level, of Pearson product-moment correlation coefficient (*r*) and the sample size (*n*). Details and required caution in the use of *r* have been reported in Bevington and Robinson [45].

On the other hand, Zou [33] reported a mass-balance approach to explain the *y*_*m*_ = (*u*/*a*)_*m*_ and *x*_*m*_ = (*v*/*b*)_*m*_ geochemical ratios (where *a*, *b*, *u*, and *v* represent major or trace elements) in SC comingled lavas as a product of a mixture of two components 1 and 2. The variation in the *y*_*m*_ and *x*_*m*_ geochemical ratios could be modeled by the hyperbolic equation (condition *a*_1_/*a*_2_≠*b*_1_/*b*_2_):

(9) $$\begin{array}{c} Ax_{m}+Bx_{m}y_{m}+Cy_{m}+D=0. \end{array}$$

In this model, the *A* to *D* coefficients have been defined as

(10a) $$\begin{array}{c} A=a_{2}b_{1}y_{2}-a_{1}b_{2}y_{1}, \end{array}$$

(10b) $$\begin{array}{c} B=a_{1}b_{2}-a_{2}b_{1}, \end{array}$$

(10c) $$\begin{array}{c} C=a_{2}b_{1}x_{1}-a_{1}b_{2}x_{2}, \end{array}$$

(10d) $$\begin{array}{c} D=a_{1}b_{2}x_{2}y_{1}-a_{2}b_{1}x_{1}y_{2}, \end{array}$$

where the geochemical ratios in the components 1 and 2 are

(11a) $$\begin{array}{c} x_{1}=\frac{v_{1}}{b_{1}}, \end{array}$$

(11b) $$\begin{array}{c} x_{2}=\frac{v_{2}}{b_{2}}, \end{array}$$

(11c) $$\begin{array}{c} y_{1}=\frac{u_{1}}{a_{1}}, \end{array}$$

(11d) $$\begin{array}{c} y_{2}=\frac{u_{2}}{a_{2}}. \end{array}$$

The proportion of the first component could be estimated by

(12) $$\begin{array}{c} f_{1}=\frac{{-a}_{2}y_{m}+a_{2}y_{2}}{\left(a_{1}-a_{2}\right)y_{m}-a_{1}y_{1}+a_{2}y_{2}}. \end{array}$$

In this work, the scheme described by Zou ([33], model B) was applied to evaluate the mixing/mingling process in the SC northern sector. All calculations of mixing models were carried out using the STATISTICA for Windows software.

## 5. Results

**Figure 3:**
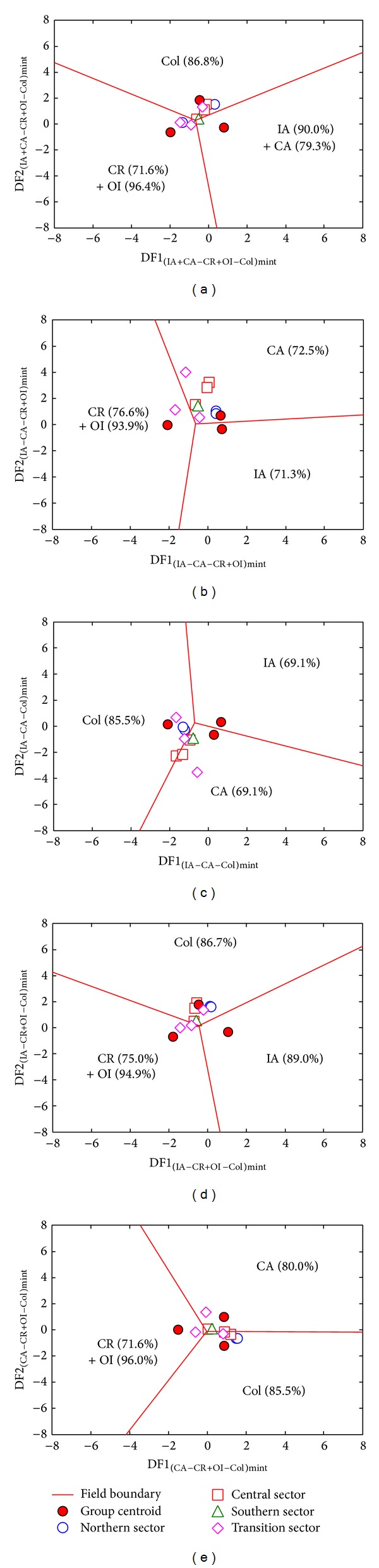
Discriminant-function multidimensional diagrams [21], based on ln-transformed ratios of major elements, for the tectonic discrimination of intermediate Sierra de las Cruces rocks. Tectonic settings: IA: island arc, CA: continental arc, CR: continental rift, OI: ocean island, and Col: collision. The symbols are explained as inset in (a). In (a), five groups are represented as three groups by combining IA and CA as IA + CA and CR and OI as CR + OI. The other four diagrams ((b)–(e)) are for three groups at a time. The subscript mint refers to the set of multidimensional diagrams based on ln-transformed major element (m) ratios for intermediate (int) magmas. Filled circles display the compositional centroid for each tectonic setting. The percentages in each field are the discrimination effectivity. The thick lines represent equal probability discrimination boundaries in all diagrams. The coordinates of the field boundaries and additional information are reported in [21].

**Figure 4:**
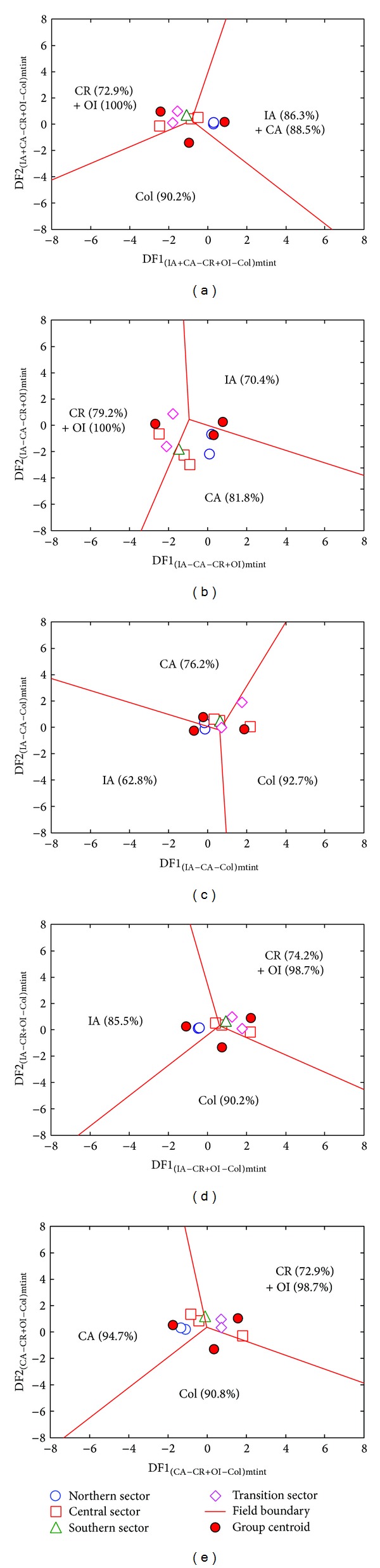
Discriminant-function multidimensional diagrams based on ln-transformed ratios of immobile major and trace elements for tectonic discrimination of intermediate Sierra de las Cruces magmas. The symbols are explained as inset in (a); more details are in Figure 3. The subscript “mtint” in axis names refers to major (m) and trace (t) element ratios for intermediate (int) magmas.

**Figure 5:**
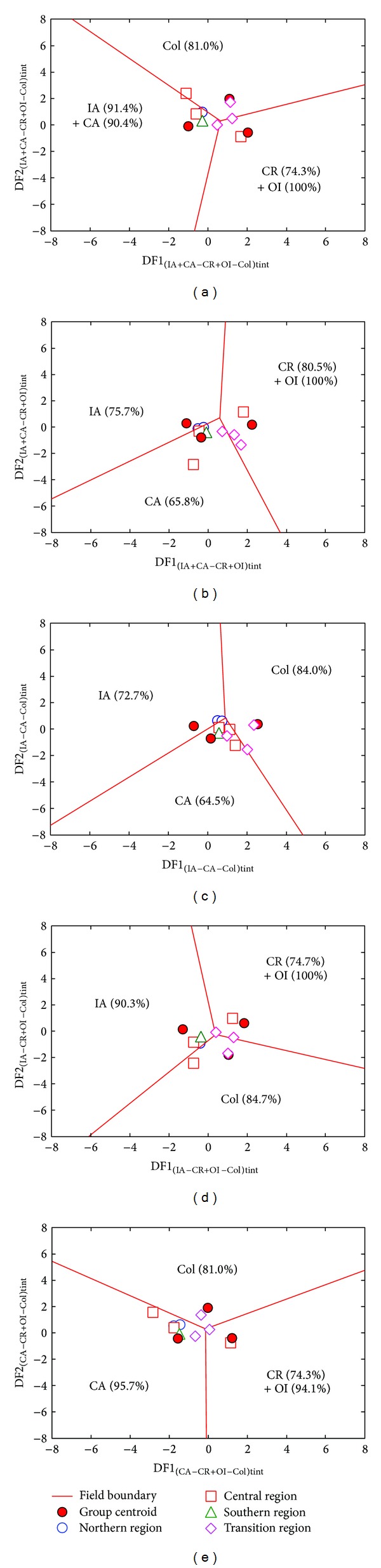
Discriminant-function multidimensional diagrams based on ln-transformed ratios of immobile trace elements for tectonic discrimination of intermediate Sierra de las Cruces magmas. The symbols are explained as inset in (a); more details are in Figure 3. The subscript “tint” in axis names refers to trace (t) element ratios for intermediate (int) magmas.

Ten samples of SC database proved to be intermediate magmas. The set of major element based diagrams (*n* = 10; Table 4 and Figure 3) showed a collisional setting with total percent probability value (% prob) of about 45.8%. However, immobile major and trace element based diagrams (*n* = 9; Table 4 and Figure 4) indicated a within-plate regime, although with a relatively low % prob of only about 38.1. Unlike other sets of diagrams, a continental arc setting can be inferred from those based on immobile trace elements (*n* = 10; % prob = 39.7; Table 4 and Figure 5). It is important to note that intermediate samples from southern and transition sectors (1.9 to 0.5 Ma) represent the main contribution to the collisional and within-plate settings.

**Figure 6:**
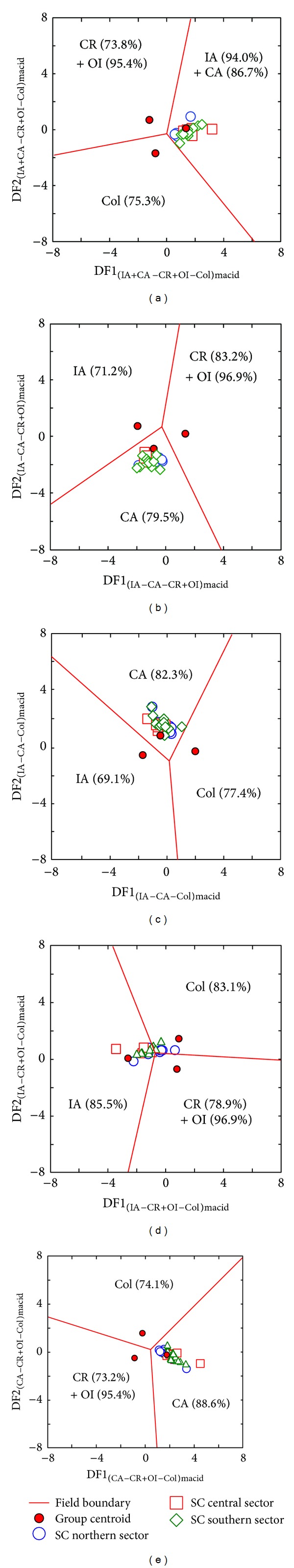
Discriminant-function multidimensional diagrams [22], based on ln-transformed ratios of major elements, for the tectonic discrimination of acid Sierra de las Cruces rocks. Tectonic settings: IA: island arc, CA: continental arc, CR: continental rift, OI: ocean island, and Col: collision. The symbols are explained as inset in (a). In (a), five groups are represented as three groups by combining IA and CA as IA + CA and CR and OI as CR+OI. The other four diagrams ((b)–(e)) are for three groups at a time. The subscript “macid” refers to the set of multidimensional diagrams based on ln-transformed major element (m) ratios for acid (acid) magmas. Filled circles display the compositional centroid for each tectonic setting. The percentages in each field are the discrimination effectivity. The thick lines represent equal probability discrimination boundaries in all diagrams. The coordinates of the field boundaries and additional information are reported in [22].

**Figure 7:**
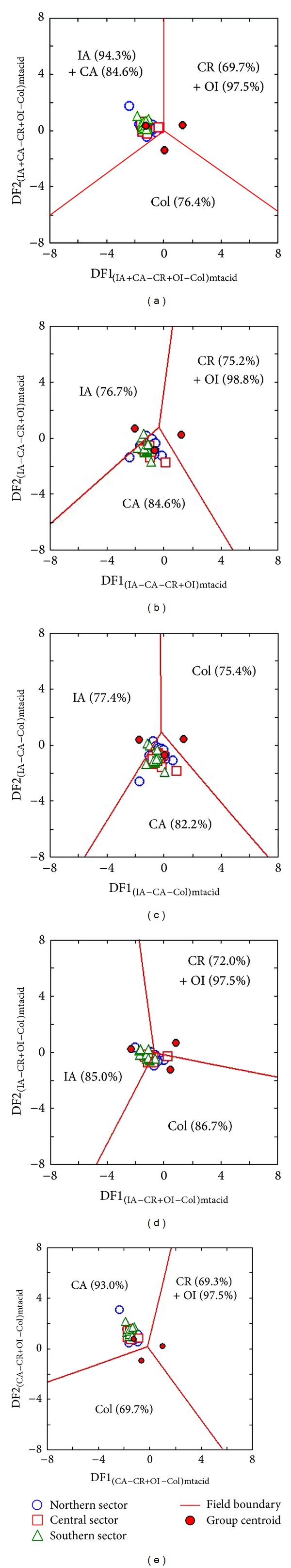
Discriminant-function multidimensional diagrams based on ln-transformed ratios of immobile major and trace elements for tectonic discrimination of acid Sierra de las Cruces magmas. The symbols are explained as inset in (a); more details are in Figure 6. The subscript “mtacid” in axis names refers to major (m) and trace (t) element ratios for acid (acid) magmas.

**Figure 8:**
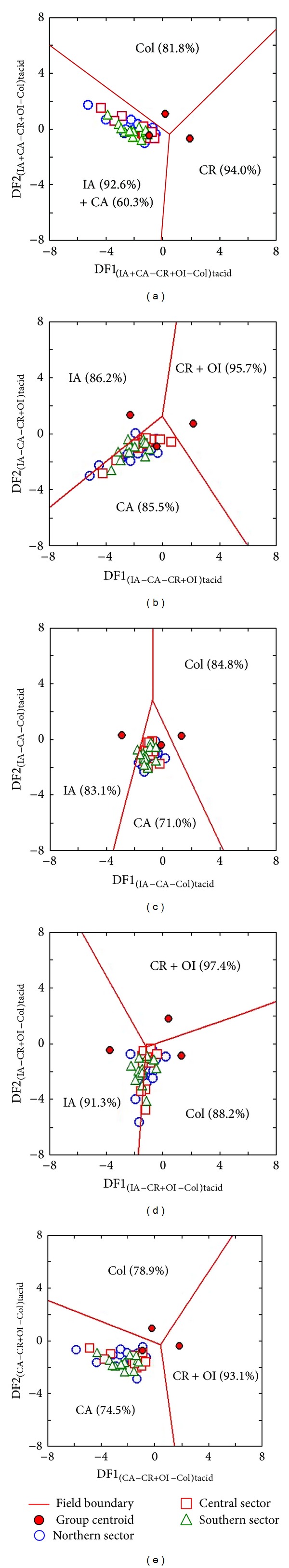
Discriminant-function multidimensional diagrams based on ln-transformed ratios of immobile trace elements for tectonic discrimination of acid Sierra de las Cruces magmas. The symbols are explained as inset in (a); more details are in Figure 6. The subscript “tacid” in axis names refers to trace (t) element ratios for acid (acid) magmas.

A relatively large number of samples (*n* = 46) from SC database proved to be of acid magma. In contrast to intermediate magmas, all diagrams indicated a subduction-related setting for the SC acid magmas, with total percent probability values for this tectonic regime of about 74.1%, 63.0%, and 68.7%, respectively, for the major, major and trace, and trace element based diagrams (Table 5 and Figures 6, 7, and 8). The results of the tectonic setting are further evaluated from discordancy and significance tests in the Discussion section below.

**Figure 9:**
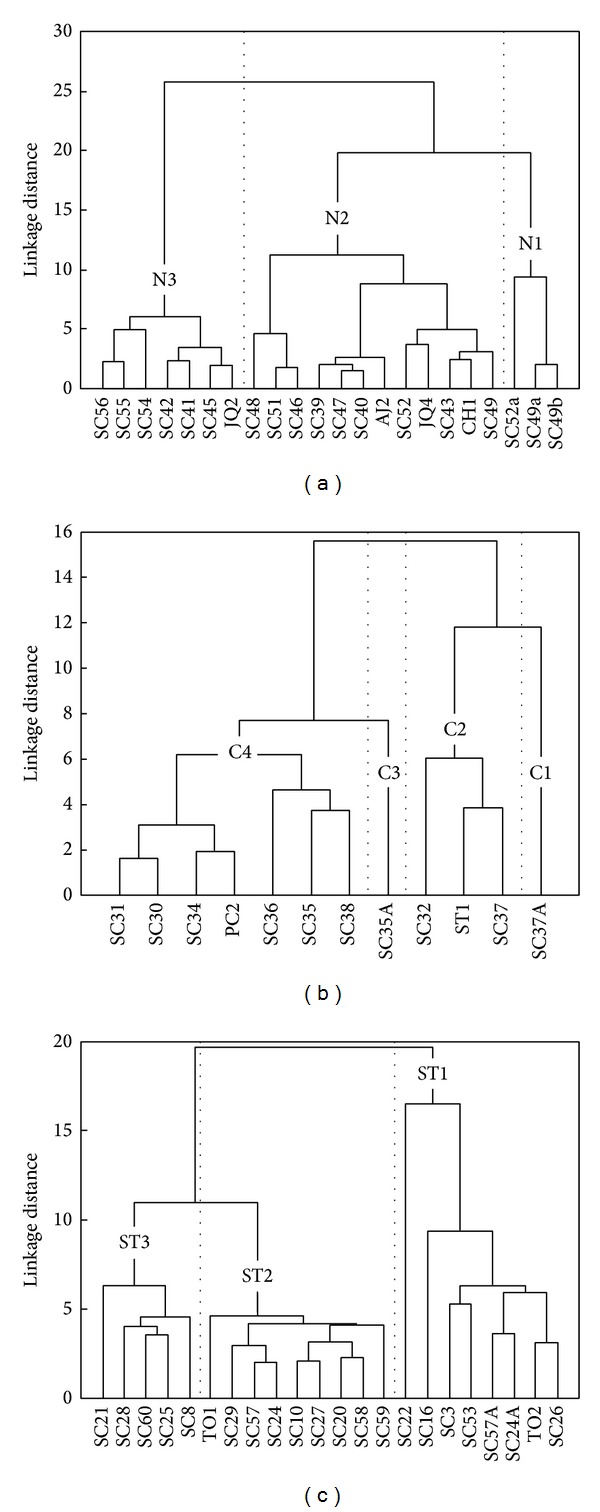
Dendrograms showing the results of the cluster analysis (considering Euclidean linkage distances) for the volcanic rocks from the (a) northern, (b) central, and (c) south + transition Sierra de las Cruces sectors.

On the other hand, the hierarchical agglomeration process was carried out for each SC sector (SCN: 22 samples; SCC: 12 samples; and SCS and SCT: 22 samples) and their results were summarized in three dendrograms with units in Euclidean values (Figures 9(a)–9(c)). The statistical parameters (mean, minimum, maximum, and standard deviation) associated with the centroid of each cluster are reported in Table 6.

**Figure 10:**
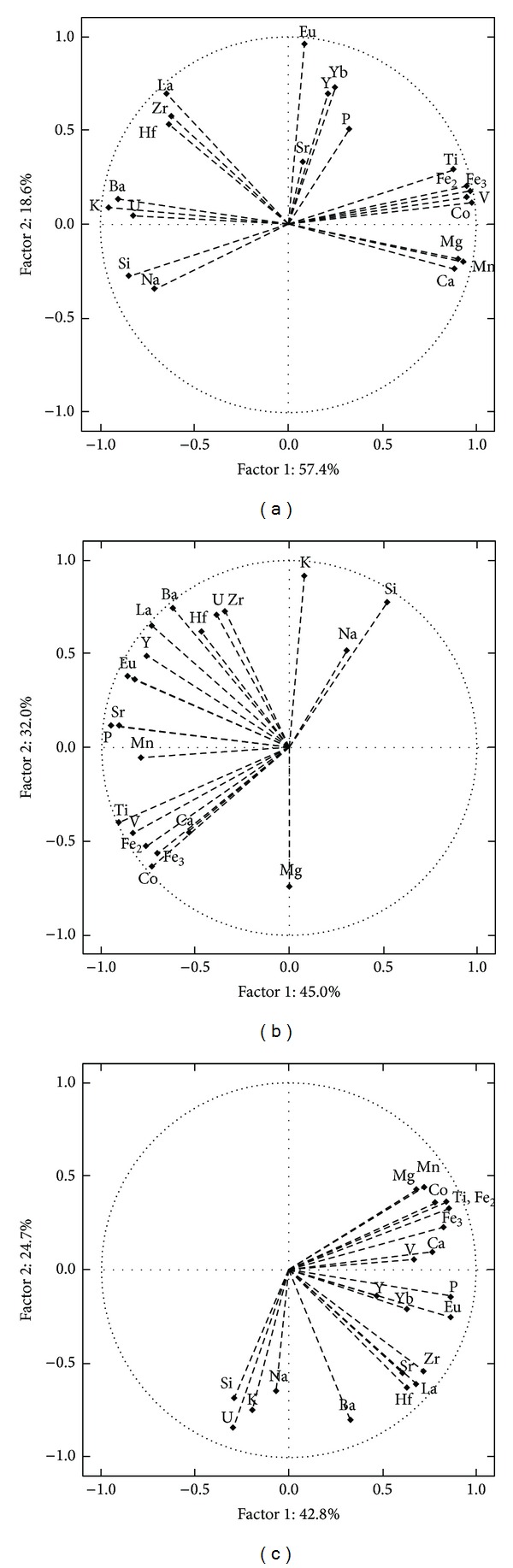
Projection of the variables on the factor-plane F2-F1 generated by principal component analysis (PCA) for the Sierra de las Cruces sectors: (a) northern, (b) central, and (c) southern + transition.

The studied rocks from northern SC sector (Table 6; Figure 9(a)) were distributed in three general clusters (N1 [13.6%], N2 [54.5%], and N3 [31.9%]). The PCA calculation indicated that the ~94.2% of geochemical variability of samples from northern SC sector could be explained by three factors. The factor F1 contributed with 57.4%, being associated with major (excepting Na and P) and transition elements; rare earth elements and yttrium ruled a contribution of 18.6% by means of the factor F2 (Figure 10(a)). The principal component F3 (a function of Na, P, and Sr) explained the 8.2% of the chemical variability.

The samples from central SC conformed four groups (C1 [8.3%], C2 [25.0%], C3 [8.3%], and C4 [58.3%]; Table 6 and Figure 9(b)). A ~94.1% of the chemical variability can be explained by means of five factors. The factor F1 (45.0%) is controlled by Si and alkali composition. A 32.0% of the compositional heterogeneity has been associated with the incompatible elements using the principal component F2 (Figure 10(b)). The factor F3 (ruled by Mg, Ca, and HFSE) contributed with a 10.6%.

The samples of SCS and SCT were agglomerated in three geochemical groups (ST1 [36.4%], ST2 [40.9%], and ST3 [22.7%]; Table 6 and Figure 9(c)). PCA calculations have revealed that a ~90% of the geochemical composition could be explained as a function of five principal components. The factor F1, associated with major elements (excepting Na and K), Co, and Eu, contributed with 42.8%. F2 factor, which represents a 24.7%, is controlled by Ba, K, and U (Figure 10(c)). An 11.9% of the chemical heterogeneity is explained by the factor F3, a variable ruled by Na, K, and V composition.

**Figure 11:**
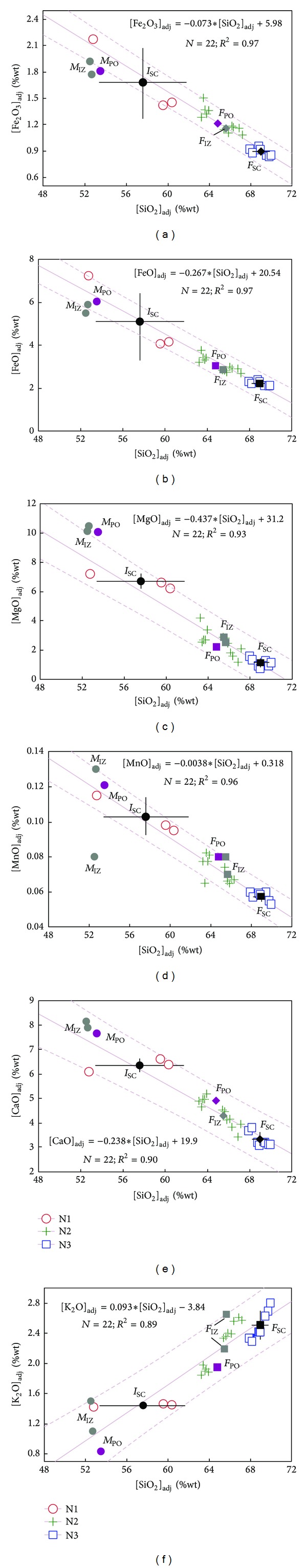
Major element Harker-type diagrams for volcanic rocks from the Sierra de las Cruces northern sector. An ordinary least-squares (OLS) regression model is included in each diagram (OLS equation; *N* is number of samples; *R* ^2^ is Pearson regression coefficient; solid line is OLS model; discontinuous lines are 95% confidence regression bands). Abbreviations for end-members in mixing/mingling models: (a) *Sierra de las Cruces*: *I*_SC_: intermediate and *F*_SC_: felsic; (b) *Iztaccíhuatl volcano* [31]: *M*_IZ_: mafic and *F*_IZ_: felsic; (c) *Popocatépetl volcano* [32]: *M*_PO_: mafic and *F*_PO_: felsic.

**Figure 12:**
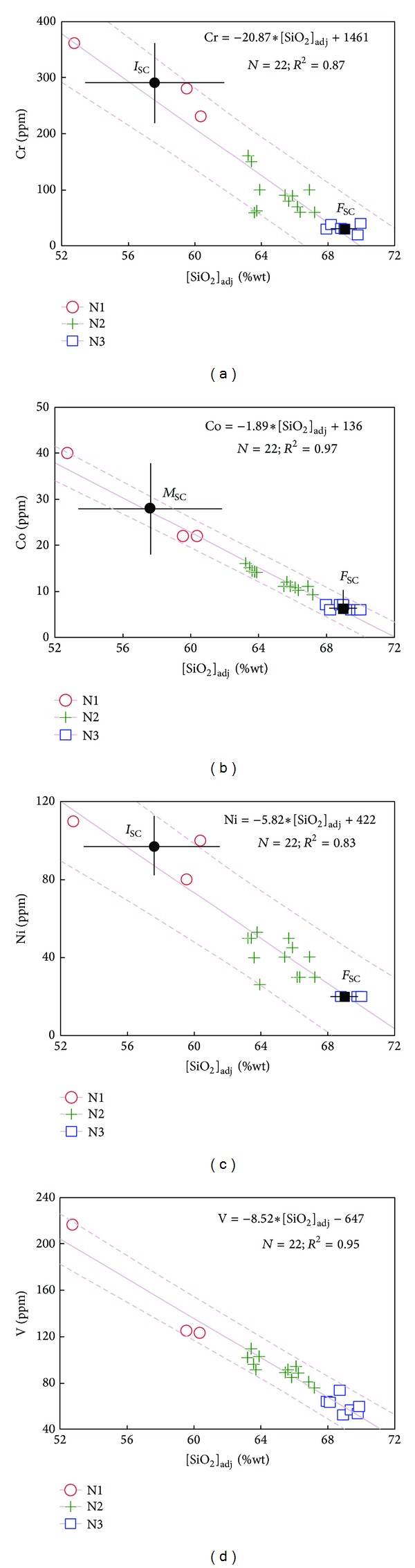
Trace element Harker-type diagrams for volcanic rocks from the Sierra de las Cruces northern sector. OLS regression models as those presented in Figure 11.

The mass-balance approach for magma mixing (model A) used by Nixon [31] was applied to the geochemical data from SC northern sector (i.e., intermediate N1 cluster interacting with felsic N3 group resulting in N2 comingled lavas). The mixing analysis was essentially limited to [SiO_2_]_adj_, [Fe_2_O_3_]_adj_, [FeO]_adj_, [MnO]_adj_, [MgO]_adj_, [CaO]_adj_, [K_2_O]_adj_, Co, Cr, Ni, and V, since all these constituents exhibit a statistically significant linear coherence in Harker diagrams (*r* = 0.89–0.98; *n* = 22; statistically significant at 99% confidence level; Figures 11 and 12) and have relatively small concentration ranges in felsic N3 end-member (Table 6).

**Figure 13:**
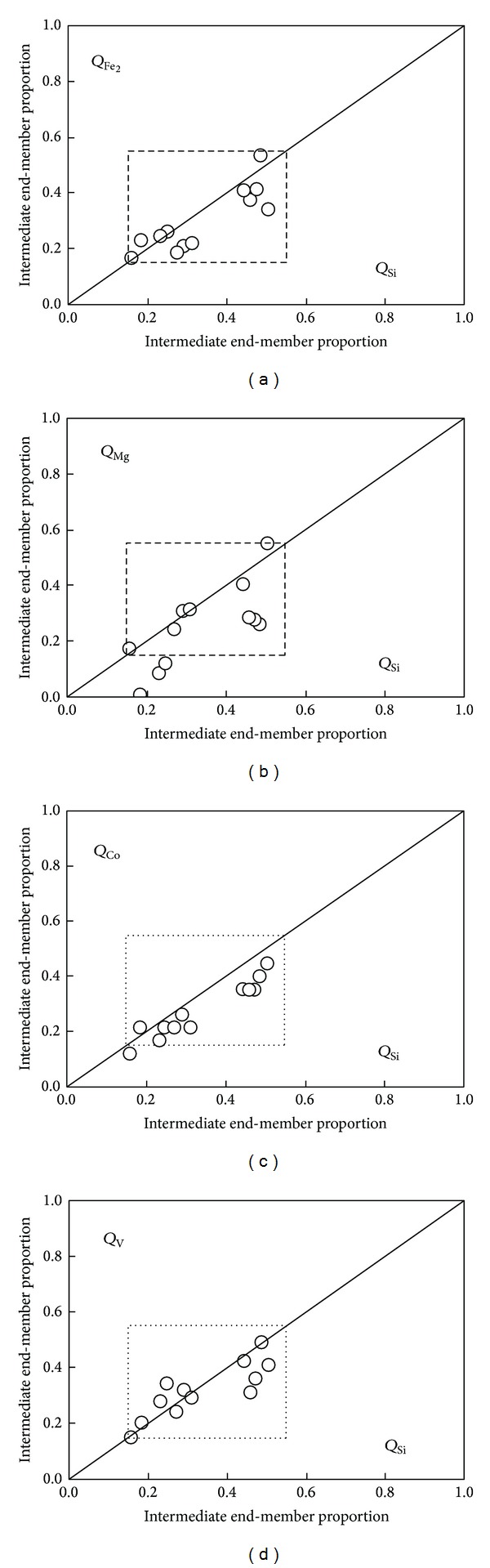
Mean proportions of mafic end-members in the comingled lavas from the Sierra de las Cruces, calculated using the mass-balance equation *Q*_*A*_^*i*^ = |*C*_*M*_^*i*^ − *C*_*B*_^*i*^|/|*C*_*A*_^*i*^ − *C*_*B*_^*i*^| [31] for [SiO_2_]_adj_, [FeO]_adj_, [MgO]_adj_, Co, and V. Proportions determined using [SiO_2_]_adj_ are plotted against those obtained using the other constituents. The diagonal line indicates perfect agreement between results.

**Figure 14:**
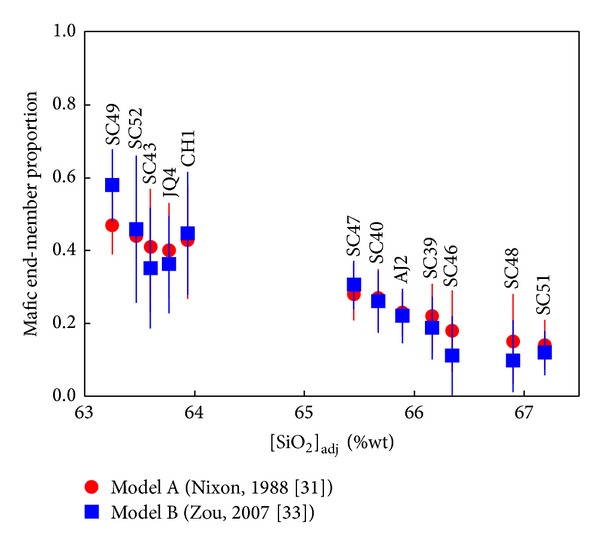
Mean ± one standard deviation of intermediate end-member proportions (N1) in the comingled lavas (N2) from the Sierra de las Cruces northern sector versus [SiO_2_]_adj_, produced by the incomplete mixing of N1 and N3 end-members: (a) red filled circle and line calculated (*n* = 11) from the mass-balance approach proposed by Nixon [31] and (b) blue filled square and line calculated (*n* = 12) from the mass-balance approach proposed by Zou [33].

The proportion of the intermediate N1 end-member in each N2 mixed lava was calculated using (7) and the average composition of the intermediate (*I*_SC_) and felsic (*F*_SC_) end-members. Calculated proportions exhibit internal consistency for majority of the chemical components (Figure 13). For each sample, the estimated proportions display a Gaussian distribution (their normality behavior was proved by a Schapiro-Wilks test), covering between ~15 and 47% in average proportion of the andesitic N1 end-member (Figure 14).

**Figure 15:**
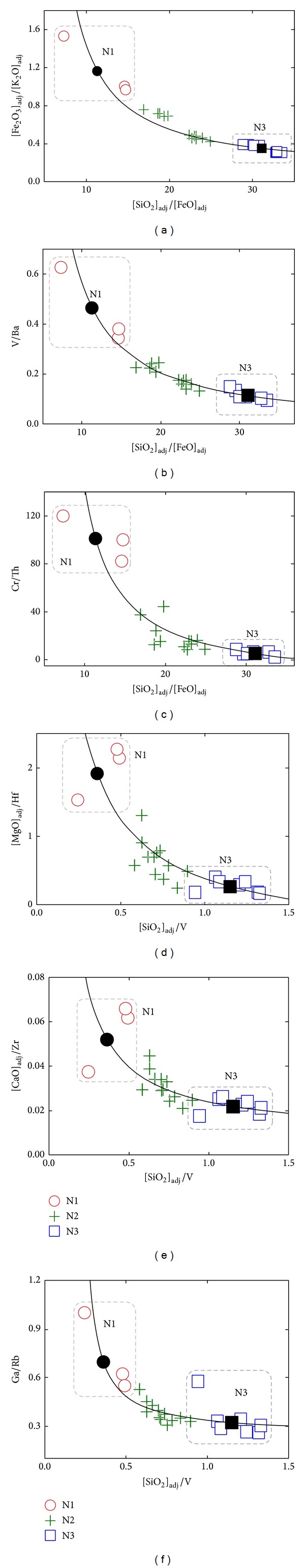
Geochemical ratio-ratio diagrams of the Sierra de las Cruces northern sector that include hyperbolic mixing models (black solid line) between average intermediate N1 lavas (black filled circle, *I*_SC_) and average felsic N3 lavas (black filled square, *F*_SC_): (a) [Fe_2_O_3_]_adj_/[K_2_O]_adj_ − [SiO_2_]_adj_/[FeO]_adj_; (b) V/Ba − [SiO_2_]_adj_/[FeO]_adj_; (c) Cr/Th − [SiO_2_]_adj_/[FeO]_adj_; (d) [MgO]_adj_/Hf − [SiO_2_]_adj_/V; (e) [CaO]_adj_/Zr − [SiO_2_]_adj_/V; (f) Ga/Rb − [SiO_2_]_adj_/V. Hyperbolic mixing equations, generated following the mass-balance approach by Zou [33], are reported in Table 7.

**Figure 16:**
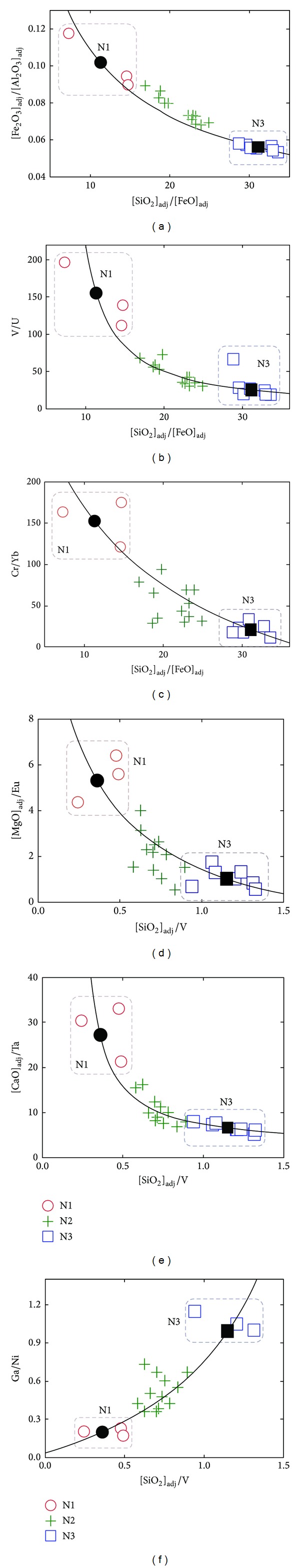
Geochemical ratio-ratio diagrams of the Sierra de las Cruces northern sector that include hyperbolic mixing models (black solid line) between average intermediate N1 lavas (black filled circle, *I*_SC_) and average felsic N3 lavas (black filled square, *F*_SC_): (a) [Fe_2_O_3_]_adj_/[Al_2_O_3_]_adj_ −  [SiO_2_]_adj_/[FeO]_adj_; (b) V/U − [SiO_2_]_adj_/[FeO]_adj_; (c) Cr/Yb − [SiO_2_]_adj_/[FeO]_adj_; (d) [MgO]_adj_/Eu − [SiO_2_]_adj_/V; (e) [CaO]_adj_/Ta − [SiO_2_]_adj_/V; (f) Ga/Ni − [SiO_2_]_adj_/V. Hyperbolic mixing equations, generated following the mass-balance approach by Zou [33], are reported in Table 7.

On the other hand, the mixing model B [33] was applied to lavas of the northern SC sector. The coefficients *A* to *D* ((10a)–(10d)) of the hyperbolic mixing equation (9) were established for twelve geochemical ratio-ratio *u*/*a* – *v*/*b* systems (Table 7): (1)  *u*/*a*: [Fe_2_O_3_]_adj_/[K_2_O]_adj_, [Fe_2_O_3_]_adj_/[Al_2_O_3_]_adj_, V/Ba, V/U, Cr/Th, and Cr/Yb – *v*/*b*: [SiO_2_]_adj_/[FeO]_adj_; (2)  *u*/*a*:[MgO]_adj_/Eu, [MgO]_adj_/Hf, [CaO]_adj_/Ta, [CaO]_adj_/Zr, Ga/Ni, and Ga/Rb – *v*/*b*: [SiO_2_]_adj_/V). Figures 15 and 16 show some examples of the ratio-ratio diagrams for the SCN lavas, including the average composition of the intermediate (*I*_SC_) and felsic (*F*_SC_) end-members (black filled square and circle) and their hyperbolic mixing models (black solid line). The application of model B revealed that the percentages (100∗*f*_1_) of the component N1 in each of the comingled lavas N2 range from 11 to 58% (Figure 14). Each mean and its uncertainty were estimated from a statistic sample of twelve ratio-ratio systems displaying a Gaussian behavior (normality proved by a Schapiro-Wilks test).

## 6. Discussion

### 6.1. Tectonic Setting

The MVB (Figure 1) has been considered as a very tectonically complex zone. In the framework of the theory of plate tectonics, the origin of this volcanic province has been explained by means of the subduction of Cocos and Rivera plates under the North American plate. However, several geological, geophysical, and geochemical characteristics observed in central MVB and the entire province do not support this simple model. Particularly, a strong controversy regarding the tectonic regime has been widely documented in the literature (e.g., [29, 30, 38, 46–53]).

How to interpret the seemingly contradictory results obtained in the tectonic discrimination analysis for the SC magmas (Tables 4 and 5)? A transitional continental arc to within-plate setting can be tentatively considered as a consistent model for the central MVB. Felsic magmas display geochemical features consistent with an origin from the upper continental crust. The genesis of the majority of the Mexican crustal source rocks has been associated with continental arc regime. Afterwards, a change in the tectonic setting could be related to a relatively fast variation in the Cocos plate subduction angle.

However, the Cocos plate tectonic evolution is an issue that has not been solved. Pérez-Campos et al. [54] pointed out that the history of volcanism has been used to infer the evolving geometry of subduction. According to this model, during earlier Eocene the volcanic arc in central Mexico was nearer to the coast and parallel to the trench consistent with steep subduction. In late Eocene (30 Ma) there was a hiatus, thought to be associated with a flattening process. At 20 Ma, after a 10 Ma lull, volcanic activity resumed. At ~10 Ma, the western part of the Cocos plate separated to form Rivera plate. At about this time, the development and propagation of a tear in the subduction plate have been suggested, culminating with the lower portion of the Cocos plate breaking off. The west-east propagating volcanism along the MVB reached the longitude of Mexico City at about 7 Ma. Additionally, Peláez Gaviria et al. [55] have reported changes during the last 3.5 Ma in the plate configuration at the north of the Middle America Trench (MAT) as a result of (a) the propagation of the Pacific-Cocos Segment of the East Pacific Rise (EPR-PCS), (b) the collision of the EPR-PCS with the MAT at 1.7 Ma, and (c) the formation of the Rivera Transform.

Actually, subhorizontal subduction of Cocos plate has been inferred by Pérez-Campos et al. [54], Husker and Davis [56], and Pacheco and Singh [57] from seismic data obtained from a dense network. Particularly, the dip angle of Cocos slab decreases gradually from ~50° to 0° along the labeled Michoacan segment of the Mexican subduction zone [57]. However, this quasihorizontal subduction and a very shallow subducted slab (at most at about 40 km in depth) are not thermodynamically favorable conditions for arc-related magma generation [58].

The diminution or even cessation of arc-related volcanism observed in the south-central Andes has been related to subhorizontal subduction of the Nazca plate [59]. The SC intermediate rocks could be a volcanism generated under this complex condition of the tectonic transition to an extensional regime. Additionally, Velasco-Tapia and Verma [29] have inferred, from inverse and direct immobile trace element modeling, combined ^87^Sr/^86^Sr and ^143^Nd/^144^Nd isotopic ratios, and the use of multidimensional log-ratio discriminant-function-based diagrams, that mafic magmas from the Sierra de Chichinautzin (the post-SC volcanic event of <40 ka) were undoubtedly generated by partial melting of continental lithospheric mantle in a within-plate setting.

Although the previous studies and this work represent significant contributions to the understanding of the origin of the volcanism in the central MVB, more geological-geophysical-geochemical collaborative research is needed to clearly understand the evolution of the tectonic regime in this area and the entire MVB.

### 6.2. Application of Discordancy and Significance Tests

The acid rock data of SC were placed in two groups: *Gr1* close to the MAT (consisting of the data from the southern and transition sectors) and *Gr2* farther away from the MAT (data from the northern and central sectors). A statistical comparison of these groups was carried out using Fisher *F* test and Student's *t*-test. The results are summarized in Table 8. No statistically significant difference was observed between the two groups for any of the elements listed in Table 8 (see *true* for all elements in both one-sided and two-sided columns of Table 8). The same is true for the Nb-anomaly as well as for ratios of large-ion lithophile elements (LILE) to light rare earth elements (LREE) and LILE to high-field strength elements (HFSE) (see [38] for the importance of these ratios for subduction processes). Therefore, the negligible contribution from the subducted slab to the SC magmas can be safely inferred. The intermediate rock data were not so numerous and, therefore, are not reported here, although they confirmed the results for acid rocks.

### 6.3. Magmatic Clusters

The statistical analysis of samples from northern SC sector (Figure 9(a) and Table 6) revealed that group N1 corresponds to the intermediate magmatic enclaves (SC49A, SC49B, and SC52A). Dacitic lavas without disequilibrium features dominate the N3 group, being accompanied by some mixed lavas with similar chemical composition. These groups are widely spaced, as observed in the dendrogram, with a Euclidian linkage distance of 25. In comparison with N3 felsic magmas, the intermediate samples of N1 group have higher contents of [TiO_2_]_adj_, [Fe_2_O_3_]_adj_, [FeO]_adj_, [MnO]_adj_, [MgO]_adj_, [CaO]_adj_, and transition elements (e.g., Co and V). Cluster N2 seems to be representing the group including the majority of comingled lavas observed in this sector. It is important to note that the northern SC sector displays a relatively high density of magmatic enclaves included in felsic magmas, also showing the specimens with the higher size (reaching ~20 cm) in the entire volcanic range. This fact could be related to an increase in fault and fracture density in this direction [24], a favorable condition for magma mingling/mixing processes.

The central SC sector did not include dacitic rocks without disequilibrium features. The C1 and C3 clusters (Figure 9(b) and Table 6) represent intermediate magmatic enclaves (SC35A and SC37A). The mixed lavas were more loosely grouped in two different clusters (C2 and C4), each of them with relatively lower levels of similarity in relation to a magmatic enclave. In comparison with the northern sector, the Euclidian linkage distances are relatively tiny: C1 + C2 clusters show a separation of ~16 units in relation to C3 + C4 subgroups. The samples from southern and transition SC sectors separated into three sets (Figure 9(c) and Table 6) relating primarily to differences in [SiO_2_]_adj_, [TiO_2_]_adj_, [Fe_2_O_3_]_adj_, and [FeO]_adj_ contents. The cluster ST1 includes magmatic enclaves (with a relatively small size of ~2–4 cm) and lavas with an intermediate composition ([SiO_2_]_adj_ = 54–61%). This group shows a strong contrast in relation to the other clusters, as reflected by a Euclidean linkage distance of ~20. The majority of the dacitic mixed lavas were within the cluster ST2 ([SiO_2_]_adj_ = 63–66%), whereas dacitic lavas without disequilibrium features conformed the cluster ST3 ([SiO_2_]_adj_ = 65–69%).

### 6.4. Magma Mixing Process

Along the entire MVB, magma mixing/mingling has also been inferred as a significant mechanism in the petrologic evolution of stratovolcanoes (*Tequila* [60, 61]; *Tancítaro* [62]; *Iztaccíhuatl* [31]; *Popocatépetl* [32, 63, 64]; *Telapón* [65]), cinder cones and monogenetic fields (*Sanganguey* [66]; *Chichinautzin* [29]), or calderas (*Amealco* [67]; *La Primavera* [68]).

Particularly, seismic and gravity data have revealed the presence of partial melts at the base of the crust in the central MVB [69, 70]. These magmas might be stored at the base of the crust transferring heat to shallower crustal levels. The partial melting of the upper continental crust (depth at the base ~10 km [71]) generated dacitic magma (e.g., N3-type cluster in the SC northern sector with an *F*_SC_ average composition; Figures 9(a), 11, and 12). This relatively low-temperature magma was stored in the shallow crust. Subsequently, a small volume of andesitic magma (e.g., N1-type cluster with an *I*_SC_ average composition; Figures 11 and 12), probably generated at lower crust (depth 25–45 km [71]), intruded in the dacitic magma chamber, losing heat to the surroundings and starting to vesiculate, prior to effusion.

This interaction process between dacitic-andesitic magmas occurred continuously in the SC during a period of ~3 Ma. Mass-balance analysis (model A) for SC northern sector has showed that from ~11 to 58% of the andesitic end-member was partially mixed with the felsic magma, as observed in Q diagrams (Figure 13). Repeated injections of this andesitic magma into the dacitic magma caused mingling events in the central and the southern SC sectors.

Average value and their uncertainty for northern SC compositional poles (*I*_SC_ and *F*_SC_) have been included in the major-element Harker diagrams (Figure 11). Also, for comparison, the end-member components modeled for the magma mixing process in Popocatepetl (*M*_PO_, mafic and *F*_PO_, felsic [32]) and Iztaccihuatl (*M*_IZ_, mafic and *F*_IZ_, felsic [31]), two stratovolcanoes located behind the SC volcanic range, have been incorporated in these diagrams.

Magma mixing evaluation in SC northern sector, using the alternative approach proposed by Zou [33] (model B), resulted in hyperbolic mixing models for several ratio-ratio systems involving major and trace elements (Table 7). Mixing models (Figures 15-16) have yielded end-member compositions that are close to the samples of N1 and N3 groups. According to *F* test and *t*-test, no significant differences exist between the sample compositions and the modeled end-member compositions. Additionally, these models suggest that the comingled lava compositions can be explained by mixing N1 : N3 end-members from 0.11 : 0.89 to 0.58 : 0.42 (Figure 14). Clearly, these results are comparable to those obtained applying the mass-balance model A (Figure 14).

## 7. Conclusions

1. Statistical and mass-balance techniques have been successfully used as igneous petrological tools.
2. From multidimensional discrimination diagrams, a transitional continental arc to within-plate setting can be tentatively considered as a consistent tectonic framework for the Sierra de las Cruces volcanic range. Felsic volcanism was derived from the upper continental crust, with a continental arc affinity, whereas the intermediate magmas (spheroidal enclaves) were generated in deeper levels of the crust in an extensional setting.
3. Discordancy and significance tests have revealed that evidence does not exist of a geochemical contribution of several major and trace elements from the subducting Cocos plate to the SC magma genesis. The definitive validity of this hypothesis necessary requires, at least, a similar behavior for volatile components (water, CO_2_, SO_2_, etc.) and also fluid-linked isotopic species (e.g., Li, B). However, this information has not been available in this work.
4. A cluster analysis confirms the existence of three lithological groups in the SC: (a) dacitic lavas without disequilibrium features, (b) intermediate magmatic enclaves, and (c) comingled lavas, produced by the incomplete mixing between the other lithological clusters.
5. Mass-balance models have revealed that the chemical composition of the comingled lavas from the SC northern sector can be reproduced with ~11 to 58% of the andesitic end-member.

## Figures and Tables

**Table 1:** Petrographic information of the Sierra de las Cruces volcanic rocks^a^.

Sample	Locality	Lat. (N)°	Long. (W)°	Texture	Phenocrysts						Groundmass texture	Rock	Disequilibrium evidence			
					Ol	Opx	Cpx	Plg	Qtz	Amp		Type	Ol + Qtz	Qtz-R	Plg-N + S	E
SC03	Cantimplora	99°14′34′′	19°11′35′′	VP	94			6			M	I				
SC16	Rancho Agustín	99°19′40′′	19°11′30′′	P	70	2	2	6	20		M	IDE	*⨀*	*⨀*	*⨀*	
SC22	Volcán Negro	99°23′06′′	19°10′00′′	VT	85		15				T	I				
SC46	Los Puercos	99°28′04′′	19°31′36′′	P			4	58	20	18	M	FDE			*⨀*	
SC51	Cerro Prieto	99°16′55′′	19°18′42′′	P		3	3	59	10	25	V	FDE		*⨀*	*⨀*	
SC52	S Miguel Tecpan	99°24′33′′	19°31′12′′	VP			27	41	4	28	M	FDE		*⨀*	*⨀*	*⨀*
SC52a	S Miguel Tecpan	99°24′33′′	19°31′12′′	VP				23		77	M	ME				
SC53	Santiago	99°16′53′′	19°15′48′′	VP				69	7	24	V	FDE		*⨀*	*⨀*	
SC58	Garambullos	99°15′56′′	19°15′03′′	P		3	2	58	6	31	V	FDE		*⨀*	*⨀*	
SC60	Quellamecal	99°14′25′′	19°10′33′′	P		5	3	62		30	V	F				

^a^Modal data are percentages of phenocrysts + microphenocrysts calculated on a vesicle and groundmass free basis. Texture: P: porphyritic, VP: vesicular-porphyritic, and VT: vesicular trachytic. Groundmass represents 60–90% of the rocks. Groundmass texture: M: microlithic, T: trachytic, and V: vitreous. Phenocrysts: Ol: olivine, Opx: orthopyroxene, Cpx: clinopyroxene, Plg: plagioclase, Qtz: quartz, and Amp: amphibole. Rock types: I: intermediate magmas without disequilibrium evidence, F: felsic magmas without disequilibrium evidence, IDE: intermediate comingled lava, FDE: felsic comingled lava, and ME: magmatic enclave. Disequilibrium evidences: Ol + Qtz: olivine + quartz, Qtz-R: quartz with a reaction rim, Plg-N + S: plagioclases with normal and sieved texture, and E: ellipsoidal chilled andesitic enclave.

**Table 2:** Major element composition (% m/m) and CIPW norm for the volcanic rocks from the Sierra de las Cruces range^a^.

Sample	SC03	SC16	SC22	SC46	SC51	SC52	SC52a	SC53	SC58	SC60

Sector	SCT	SCT	SCT	SCN	SCN	SCN	SCN	SCS	SCS	SCS

TAS	D	A	BTA	D	D	D	BA	A	D	D

Major-element measured composition (% m/m)										
SiO_2_	60.88	54.64	53.44	64.29	65.91	60.73	48.34	54.81	63.21	66.71
TiO_2_	0.981	0.912	1.542	0.661	0.583	0.785	1.636	1.128	0.695	0.582
Al_2_O_3_	16.72	19.80	15.51	15.98	15.24	16.10	16.91	18.99	16.89	15.85
Fe_2_O_3_^t^	4.57	5.43	8.66	4.28	4.00	5.43	9.35	6.67	5.00	3.90
MnO	0.099	0.092	0.136	0.065	0.061	0.062	0.105	0.103	0.073	0.062
MgO	6.12	2.23	6.71	1.52	2.02	2.43	6.59	2.34	1.37	1.68
CaO	5.57	2.66	7.43	3.69	3.87	4.44	5.56	4.59	4.30	3.90
Na_2_O	2.97	3.47	3.98	4.07	4.01	4.01	2.36	3.68	4.40	4.56
K_2_O	0.80	0.80	1.53	2.50	2.52	1.89	1.30	0.84	1.68	2.23
P_2_O_5_	0.14	0.22	0.63	0.18	0.18	0.21	0.19	0.26	0.13	0.17
LOI	0.60	8.95	0.11	2.70	2.30	3.01	7.59	7.01	3.06	0.24
Total	**99.430**	**99.204**	**99.678**	**99.906**	**100.704**	**99.097**	**99.931**	**100.421**	**100.808**	**99.884**

CIPW norm										
Q	18.730	23.358	—	20.600	21.530	16.476	5.223	16.302	18.988	20.458
Or	4.621	5.260	9.142	15.123	15.182	11.672	8.386	5.342	10.194	13.261
Ab	25.520	32.678	34.050	35.539	34.591	35.463	21.797	33.517	38.230	38.839
An	27.132	13.089	20.155	17.677	16.451	21.265	28.762	22.684	21.039	16.298
C	1.230	9.925	—	0.330	—	—	2.145	4.633	0.333	—
Di	—	—	10.431	—	1.568	0.264	—	—	—	1.685
Hy	18.933	11.093	18.543	7.305	7.560	10.617	26.668	12.075	7.579	6.444
Ol	—	—	0.202	—	—	—	—	—	—	—
Mt	1.612	2.099	3.040	1.695	1.564	2.178	3.146	2.494	1.970	1.506
Il	1.892	1.928	2.961	1.295	1.128	1.557	3.392	2.306	1.356	1.113
Ap	0.329	0.568	1.476	0.431	0.424	0.507	0.480	0.649	0.308	0.396
Mg-v	77.719	51.684	66.871	48.905	57.634	54.66	63.940	47.752	42.473	53.718
FeO^t^/MgO	0.672	2.191	1.161	2.533	1.782	2.01	1.277	2.565	3.283	2.089

^a^TAS: rock classification following the Le Bas et al [36] scheme. A: andesite, BA: basaltic andesite, BTA: basaltic trachyandesite, and D: dacite.

Adjusted composition (% m/m) and CIPW norm calculated applying SINCLAS program [14, 15]. Mg-v = 100∗Mg^+2^/(Mg^+2^ + 0.9∗[Fe^+2^ + Fe^+3^]), atomic; Fe^+2^ and Fe^+3^ calculated from adjusted FeO and Fe_2_O_3_ following Middlemost [34].

**Table 3:** Trace element composition (ppm) for the volcanic rocks from the Sierra de las Cruces range.

Sample	SC03	SC16	SC22	SC46	SC51	SC52	SC52a	SC53	SC58	SC60

Sector	SCT	SCT	SCT	SCN	SCN	SCN	SCN	SCS	SCS	SCS

TAS	D	A	BTA	D	D	D	BA	A	D	D

La	14.1	20.0	34.8	23.4	25.2	24.1	16.2	18.8	11.1	17.1
Ce	31.2	47.9	77.8	41.2	40.6	38.2	40.9	41.2	21.8	34.1
Pr	4.03	7.28	10.10	6.54	6.12	6.71	5.19	5.14	2.99	4.35
Nd	17.1	29.7	42.9	26.5	24.7	28.4	24.3	21.6	12.6	17.4
Sm	3.9	6.1	9.0	5.4	4.8	5.6	5.5	4.6	2.9	3.6
Eu	1.24	1.77	2.67	1.52	1.37	1.66	1.65	1.57	1.08	1.10
Gd	3.9	6.2	8.0	4.4	4.3	5.0	5.3	4.6	2.9	3.3
Tb	0.6	0.9	1.1	0.7	0.7	0.7	0.8	0.7	0.5	0.5
Dy	3.3	5.2	5.7	3.8	3.7	4.0	4.6	3.9	2.6	2.7
Ho	0.7	1.0	1.0	0.7	0.7	0.8	0.9	0.8	0.5	0.5
Er	1.9	2.7	2.9	2.0	2.1	2.2	2.5	2.2	1.5	1.6
Tm	0.28	0.37	0.42	0.30	0.30	0.30	0.36	0.32	0.22	0.22
Yb	1.7	1.8	2.5	2.0	1.9	1.9	2.2	1.9	1.4	1.4
Lu	0.24	0.32	0.40	0.30	0.30	0.29	0.33	0.29	0.23	0.21
Sc	15		19	11	9	15	37	18	13	8
V	102	39	150	88	75	109	216	119	59	51
Cr	246	28	260	60	60	150	360	150	170	40
Co	18	10	29	10	9	15	40	21	15	9
Ni	88		110	30	30	50	110	80	50	20
Cu	21	94	30	10	20	20	60		20	20
Ga	13	22	20	18	20	21	22	24	21	21
Rb	13	3	28	59	61	40	22	6	38	58
Sr	380	303	763	521	502	582	445	569	454	368
Y	20	32	28	20	21	19	22	21	15	18
Zr	136	156	237	156	160	158	162	194	143	149
Nb	6.0	5.4	17.0	4.0	4.0	3.0	3.0	8.0	13.0	4.0
Cs			2.1	2.8	2.7	1.1			1.2	2.1
Ba	276	412	648	542	571	481	344	660	388	481
Hf	3.4	4.4	5.4	4.2	4.2	4.4	4.7	4.8	3.8	4.2
Ta	0.40	0.33	1.10	0.5	0.5	0.30	0.20	0.7	0.3	0.6
Pb	72		11	11	14	11	23	10	9	11
Th	1.8	3.0	4.1	6.7	6.7	4.0	3.0	5.0	3.4	8.2
U	0.6	1.3	1.2	2.6	2.5	1.6	1.1	1.1	1.4	3.1

**Table 4:** Tectonic discrimination analysis of intermediate magmas from the Sierra de las Cruces using multidimensional diagrams [21].

Figure name^a^	Figure type^a^	Total number of samples	Number of discriminated samples				
			Arc			Within-plate	Collision
			IA + CA $\left[\overset{-}{x}\pm s\right]$ (*p*_IA+CA_)^b^	IA $\left[\overset{-}{x}\pm s\right]$ (*p*_IA_)^b^	CA $\left[\overset{-}{x}\pm s\right]$ (*p*_CA_)^b^	CR + OI $\left[\overset{-}{x}\pm s\right]$ (*p*_CR+OI_)^b^	Col $\left[\overset{-}{x}\pm s\right]$ (*p*_Col_)^b^
Major elements	1 + 2-3 + 4-5	10	0	—	—	3 [0.27 ± 0.31] (0.009–0.767)	**7** [0.52 ± 0.30] (0.152–0.848)
	1-2-3 + 4	10	—	0	**8** [0.60 ± 0.29] (0.108–0.944)	2 [0.25 ± 0.32] (0.015–0.858)	—
	1-2-5	10	—	0	**5** [0.44 ± 0.24] (0.068–0.946)	—	**5** [0.45 ± 0.23] (0.030–0.861)
	1-3 + 4-5	10	—	0	—	3 [0.28 ± 0.32] (0.011–0.802)	**7** [0.62 ± 0.32] (0.147–0.959)
	2-3 + 4-5	10	—	—	4 [0.39 ± 0.17] (0.159–0.778)	1 [0.19 ± 0.21] (0.010–0.650)	**5** [0.42 ± 0.26] (0.032–0.756)
All major element based diagrams	{∑*n*} {∑prob} [% prob]	{50}	{0} {0.0} [—]	{0} {0.0} [0%]	{17} {16.552} [37.9%]	{9} {7.112} [16.3%]	{**24**} {20.061} [**45.8%**]

Major and trace elements	1 + 2-3 + 4-5	9	3 [0.34 ± 0.33] (0.005–0.846)	—	—	**6** [0.49 ± 0.35] (0.016–0.886)	0
	1-2-3 + 4	9	—	1 [0.19 ± 0.20] (0.004–0.584)	2 [0.27 ± 0.20] (0.012–0.545)	**6** [0.55 ± 0.38] (0.012–0.984)	—
	1-2-5	9	—	1 [0.27 ± 0.17] (0.016–0.518)	**6** [0.42 ± 0.16] (0.042–0.567)	—	2 [0.31 ± 0.30] (0.045–0.942)
	1-3 + 4-5	9	—	3 [0.31 ± 0.32] (0.005–0.816)	—	**6** [0.50 ± 0.33] (0.019–0.855)	0
	2-3 + 4-5	9	—	—	**5** [0.51 ± 0.38] (0.002–0.921)	4 [0.37 ± 0.31] (0.012–0.855)	0
All major and trace element based diagrams	{∑*n*} {∑prob} [% prob]	{45}	{3} {3.037} [—]	{5} {8.023} [17.8%]	{13} {12.649} [28.1%]	{**22**} {17.163} [**38.1%**]	{2} {7.193} [16.0%]

Trace elements	1 + 2-3 + 4-5	10	**6** [0.47 ± 0.33] (0.019–0.822)	—	—	2 [0.20 ± 0.32] (0.001–0.961)	2 [0.33 ± 0.27] (0.016–0.933)
	1-2-3 + 4	10	—	0	**7** [0.50 ± 0.25] (0.026–0.938)	3 [0.27 ± 0.36] (0.001–0.958)	—
	1-2-5	10	—	1 [0.21 ± 0.16] (0.009–0.467)	**7** [0.49 ± 0.18] (0.050–0.676)	—	2 [0.30 ± 0.26] (0.090–0.942)
	1-3 + 4-5	10	—	**5** [0.41 ± 0.32] (0.012–0.785)	—	3 [0.21 ± 0.32] (0.002–0.940)	2 [0.39 ± 0.29] (0.026–0.941)
	2-3 + 4-5	10	—	—	**7** [0.62 ± 0.35] (0.026–0.925)	2 [0.17 ± 0.31] (0.001–0.958)	1 [0.20 ± 0.24] (0.016–0.838)
All trace element based diagrams	{∑*n*} {∑prob} [% prob]	{50}	{6} {4.715} [—]	{6} {9.459} [18.9%]	{**21**} {19.856} [**39.7%**]	{10} {8.503} [17.0%]	{7} {12.178} [24.4%]

^a^“Figure name” corresponds to one of the three sets of diagrams based on major elements, immobile major and trace elements, and immobile trace elements, respectively, whereas “Figure type” gives the tectonic fields being discriminated where the tectonic group numbers are as follows: 1—IA (island arc), 2—CA (continental arc), 3—CR (continental rift) and 4—OI (ocean island) together as within-plate, and 5—Col (collision); *x* ± *s*—mean ± one standard deviation of the probability estimates for all samples discriminated in a given tectonic setting; these are reported in [].

^b^Probability estimates for different tectonic groups are summarized after the number of discriminated samples as follows: [*p*_IA+CA_]—range of probability values estimated for IA + CA combined setting, [*p*_IA_]—for IA, [*p*_CA_]—for CA, [*p*_CR+OI_]—for CR + OI, and [*p*_Col_]—for Col. Boldface font shows the expected or more probable tectonic setting; the final row gives a synthesis of results as {∑*n*}  {∑prob}  [%prob] where one has the following: {∑*n*}—number of samples plotting in all five diagrams which are reported in the column of total number of samples whereas the sum of samples plotting in a given tectonic field is reported in the respective tectonic field column, {∑prob}—sum of probability values for all samples plotting in a given tectonic field which are reported in the respective tectonic field column, and [%prob]—total probability of a given tectonic setting expressed in percent after assigning the probability of IA + CA to IA and CA using weighing factors. For details of principles, equations, and application rules of the multidimensional diagrams see [21].

**Table 5:** Tectonic discrimination analysis of felsic magmas from the Sierra de las Cruces using multidimensional diagrams [22]^a^.

Figure name	Figure type	Total number of samples	Number of discriminated samples				
			Arc			Within-plate	Collision
			IA + CA $\left[\overset{-}{x}\pm s\right]$ (*p*_IA+CA_)	IA $\left[\overset{-}{x}\pm s\right]$ (*p*_IA_)	CA $\left[\overset{-}{x}\pm s\right]$ (*p*_CA_)	CR + OI $\left[\overset{-}{x}\pm s\right]$ (*p*_CR+OI_)	Col $\left[\overset{-}{x}\pm s\right]$ (*p*_Col_)
Major elements	1 + 2-3 + 4-5	46	**46** [0.91 ± 0.08] (0.700–0.987)	—	—	0	0
	1-2-3 + 4	46	—	0	**46** [0.92 ± 0.03] (0.815–0.969)	0	—
	1-2-5	46	—	0	**46** [0.91 ± 0.05] (0.666–0.965)	—	0
	1-3 + 4-5	46	—	**27** [0.50 ± 0.29] (0.056–0.933)	—	0	19 [0.30 ± 0.19] (0.031–0.804)
	2-3 + 4-5	46	—	—	**46** [0.95 ± 0.05] (0.767–0.995)	0	0
All major element based diagrams	{∑*n*} {∑prob} [% prob]	{230}	{46} {39.234} [—]	{27} {35.511} [17.2%]	{**138**} {152.57} [**74.1%**]	{0} {0.0} [0.0%]	{19} {17.938} [8.7%]

Major and trace elements	1 + 2-3 + 4-5	46	**46** [0.84 ± 0.33] (0.556–0.946)	—	—	0	0
	1-2-3 + 4	46	—	3 [0.22 ± 0.14] (0.010–0.651)	**43** [0.74 ± 0.13] (0.324–0.934)	0	—
	1-2-5	46	—	3 [0.20 ± 0.14] (0.007–0.671)	**43** [0.71 ± 0.12] (0.296–0.919)	—	0
	1-3 + 4-5	46	—	**33** [0.49 ± 0.22] (0.025–0.907)	—	0	13 [0.35 ± 0.16] (0.045–0.699)
	2-3 + 4-5	46	—	—	**46** [0.87 ± 0.07] (0.660–0.953)	0	0
All major and trace element based diagrams	{∑*n*} {∑prob} [% prob]	{230}	{46} {36.007} [—]	{39} {47.423} [23.4%]	{**132**} {127.66} [**63.0%**]	{0} {0.0} [0.0%]	{13} {27.658} [13.6%]

Trace elements	1 + 2-3 + 4-5	46	**46** [0.87 ± 0.08] (0.656–0.973)	—	—	0	0
	1-2-3 + 4	46	—	1 [0.14 ± 0.13] (0.003–0.560)	**45** [0.85 ± 0.12] (0.440–0.990)	0	—
	1-2-5	46	—	0	**46** [0.82 ± 0.06] (0.599–0.913)	—	0
	1-3 + 4-5	46	—	16 [0.38 ± 0.30] (0.001–0.953)	—	0	**30** [0.57 ± 0.28] (0.047–0.975)
	2-3 + 4-5	46	—	—	**46** [0.95 ± 0.05] (0.744–0.996)	0	0
All trace element based diagrams	{∑*n*} {∑prob} [% prob]	{230}	{46} {37.178} [—]	{17} {31.627} [14.9%]	{**137**} {145.56} [**68.7%**]	{0} {0.0} [0.0%]	{30} {34.795} [16.4%]

^a^For explanation see the footnote of Figure 6. For details of principles, equations, and application rules of the multidimensional diagrams see [22].

**Table:** (a)

Element	Northern SC sector (*n* = 22)											
	N1 (*n* = 3)				N2 (*n* = 12)				N3 (*n* = 7)			
	$\overset{-}{x}$	Min.	Max.	*s *	$\overset{-}{x}$	Min.	Max.	*s *	$\overset{-}{x}$	Min.	Max.	*s *
[SiO_2_]_adj_	57.6	52.77	60.38	4.2	65.1	63.25	67.19	1.5	69.0	67.94	69.98	0.8
[TiO_2_]_adj_	1.1	0.71	1.79	0.6	0.69	0.59	0.82	0.07	0.522	0.486	0.552	0.031
[Al_2_O_3_]_adj_	16.5	15.38	18.46	1.7	16.2	15.54	16.83	0.5	15.9	15.11	16.49	0.5
[Fe_2_O_3_]_adj_	1.68	1.42	2.17	0.42	1.23	1.08	1.50	0.13	0.89	0.83	0.96	0.05
[FeO]_adj_	5.1	4.05	7.23	1.8	3.08	2.70	3.76	0.33	2.22	2.08	2.39	0.11
[MnO]_adj_	0.103	0.095	0.115	0.011	0.070	0.059	0.082	0.008	0.0574	0.053	0.060	0.0026
[MgO]_adj_	6.7	6.23	7.19	0.5	2.5	1.15	4.17	0.8	1.11	0.698	1.577	0.30
[CaO]_adj_	6.34	6.07	6.59	0.26	4.4	3.41	5.18	0.5	3.31	3.08	3.79	0.30
[Na_2_O]_adj_	3.3	2.58	3.66	0.6	4.28	4.09	4.53	0.13	4.37	4.20	4.48	0.11
[K_2_O]_adj_	1.442	1.419	1.462	0.022	2.23	1.84	2.61	0.30	2.51	2.29	2.80	0.19
[P_2_O_5_]_adj_	0.164	0.143	0.207	0.037	0.193	0.151	0.292	0.036	0.1344	0.130	0.141	0.0043
La	14.0	12.3	16.2	2.0	20	13.6	34.8	6	19.8	15.1	23.6	3.2
Eu	1.26	1.03	1.65	0.34	1.32	1.04	2.15	0.32	1.07	0.91	1.25	0.12
Yb	1.90	1.60	2.20	0.30	1.76	1.26	2.70	0.40	1.52	1.16	1.70	0.22
Ba	344	329	358	15	500	414	571	60	530	472	578	50
Co	28	22	40	10	12.3	9.0	16.0	2.2	6.4	6.0	7.0	0.5
Cr	290	230	360	70	90	60	160	34	32	20	40	7
Hf	3.5	2.9	4.7	1.0	3.96	3.20	4.60	0.40	4.13	3.8	4.7	0.39
Sr	459	445	474	15	520	431	601	60	410	351	566	70
Th	2.87	2.80	3.00	0.12	5.3	3.6	6.7	1.2	6.0	3.4	8.2	1.4
U	1.03	0.90	1.10	0.12	2.10	1.40	2.60	0.44	2.4	1.10	3.00	0.6
V	160	123	216	50	92	75	109	10	60	52	73	7
Y	20.7	18	22	2.3	19	12	34	6	16.1	12.0	22.0	3.2
Zr	122	100	162	35	146	109	162	15	151	133	184	18

**Table:** (b)

Element	Central SC sector (*n* = 12)									
	C1 (*n* = 1)	C2 (*n* = 3)				C3 (*n* = 1)		C4 (*n* = 7)		
		$\overset{-}{x}$	Min.	Max.	*s *		$\overset{-}{x}$	Min.	Max.	*s *
[SiO_2_]_adj_	58.329	65.1	63.76	67.30	1.9	61.338	64.2	63.61	65.16	0.5
[TiO_2_]_adj_	1.109	0.71	0.63	0.77	0.08	0.791	0.69	0.65	0.78	0.05
[Al_2_O_3_]_adj_	17.043	16.3	15.89	16.84	0.5	17.400	16.46	15.99	17.10	0.40
[Fe_2_O_3_]_adj_	1.778	1.24	1.08	1.35	0.15	1.424	1.29	1.16	1.57	0.13
[FeO]_adj_	5.079	3.11	2.70	3.36	0.36	4.068	3.23	2.90	3.91	0.33
[MnO]_adj_	0.118	0.0783	0.0760	0.0810	0.0025	0.071	0.078	0.070	0.088	0.007
[MgO]_adj_	3.663	2.2	1.51	3.06	0.8	3.918	3.0	2.31	3.61	0.5
[CaO]_adj_	6.764	4.5	3.79	5.23	0.7	4.962	4.78	3.97	5.37	0.42
[Na_2_O]_adj_	4.290	4.35	4.27	4.49	0.12	4.270	4.36	4.16	4.62	0.17
[K_2_O]_adj_	1.497	2.163	2.136	2.180	0.023	1.592	1.77	1.66	1.95	0.11
[P_2_O_5_]_adj_	0.329	0.21	0.16	0.24	0.05	0.165	0.157	0.141	0.165	0.009
La	26.8	26.0	23.6	27.7	2.2	11.1	14.3	12.1	19.1	2.3
Eu	2.09	1.68	1.45	2.12	0.38	1.10	1.07	0.99	1.34	0.12
Yb	2.30	1.87	1.60	2.10	0.25	1.50	1.48	1.30	1.70	0.16
Ba	507	550	485	602	60	309	400	364	447	29
Co	26	13.0	10.0	15.0	2.6	17	13.9	12.0	17.0	2.0
Hf	3.90	3.73	3.60	3.90	0.15	3.20	3.59	3.40	3.70	0.11
Sr	813	600	434	683	140	453	469	451	507	21
U	1.70	1.94	1.80	2.02	0.12	1.20	1.38	1.00	1.70	0.22
V	150	89	71	103	16	100	88	81	99	7
Y	24.0	19.7	17.0	21.0	2.3	14.0	15.6	13.0	20.0	2.2
Zr	138	137.7	137	139	1.2	114	131	123	138	6

**Table:** (c)

Element	Southern and transition SC sectors (*n* = 22)											
	ST1 (*n* = 8)				ST2 (*n* = 9)				ST3 (*n* = 5)			
	$\overset{-}{x}$	Min.	Max.	*s*	$\overset{-}{x}$	Min.	Max.	*s*	$\overset{-}{x}$	Min.	Max.	*s*
[SiO_2_]_adj_	59.3	54.03	61.83	2.5	64.6	63.30	65.97	0.9	67.4	64.92	69.41	1.8
[TiO_2_]_adj_	1.09	0.84	1.56	0.23	0.72	0.63	0.92	0.09	0.61	0.54	0.66	0.05
[Al_2_O_3_]_adj_	18.1	15.68	22.04	2.1	16.7	15.76	17.75	0.6	16.26	15.95	17.04	0.44
[Fe_2_O_3_]_adj_	1.59	1.11	2.10	0.28	1.28	1.11	1.44	0.12	1.03	0.86	1.17	0.13
[FeO]_adj_	4.5	3.18	5.99	0.8	3.21	2.78	3.61	0.30	2.58	2.14	2.92	0.33
[MnO]_adj_	0.105	0.079	0.138	0.018	0.071	0.052	0.088	0.014	0.054	0.022	0.079	0.021
[MgO]_adj_	4.0	2.48	6.78	1.7	2.3	1.41	3.51	0.7	1.6	0.49	2.81	1.0
[CaO]_adj_	5.6	2.96	7.51	1.4	4.53	4.04	5.11	0.39	3.9	3.23	4.87	0.7
[Na_2_O]_adj_	4.00	3.02	4.39	0.44	4.51	4.28	4.77	0.14	4.34	4.05	4.59	0.26
[K_2_O]_adj_	1.33	0.78	2.01	0.43	1.84	1.63	2.09	0.15	2.19	1.94	2.35	0.15
[P_2_O_5_]_adj_	0.27	0.14	0.64	0.16	0.17	0.11	0.25	0.05	0.155	0.135	0.177	0.018
La	18	11.5	34.8	7	14.5	11.1	18.2	2.2	19.0	16.9	25.2	3.5
Eu	1.6	1.13	2.67	0.5	1.13	1.04	1.34	0.09	1.24	1.10	1.49	0.16
Yb	2.0	1.3	2.7	0.5	1.52	1.01	2.30	0.34	1.74	1.40	2.10	0.30
Ba	420	276	660	150	416	369	471	37	469	434	499	25
Co	19	10	29	5	12.9	10.0	20.0	3.3	8.8	5.0	12.0	2.9
Hf	4.2	3.4	5.4	0.7	3.68	3.30	4.10	0.24	3.90	3.60	4.20	0.28
Sr	500	303	763	130	474	416	533	40	410	364	495	50
U	1.04	0.60	1.32	0.28	1.50	0.80	2.00	0.34	2.0	1.5	3.1	0.6
V	110	39	150	33	83	59	96	12	69	51	84	12
Y	23	13.0	32.2	6	14.9	11.0	17.0	1.8	22	16	36	8
Zr	162	129	237	38	136	125	149	8	148	126	164	14

**Table 7:** *A*–*D* coefficients of hyperbolic Equations (10a)–(10d) for magma mixing between N1 and N3 end-members (northern Sierra de las Cruces sector), generated applying the mass-balance model by Zou [33].

Ratio-ratio system	*y*-axis	*x*-axis	Hyperbolic mixing equation coefficients			
			*A*	*B*	*C*	*D*
1	[Fe_2_O_3_]_adj_/[K_2_O]_adj_	[SiO_2_]_adj_/[FeO]_adj_	0.81	−9.60	45.1	64.7
2	[Fe_2_O_3_]_adj_/[Al_2_O_3_]_adj_	[SiO_2_]_adj_/[FeO]_adj_	0.81	−44.5	−223	64.7
3	V/Ba	[SiO_2_]_adj_/[FeO]_adj_	−49.2	−1939	6792	7584
4	V/U	[SiO_2_]_adj_/[FeO]_adj_	−49.2	−9.95	67.2	7584
5	Cr/Th	[SiO_2_]_adj_/[FeO]_adj_	−481	−24.2	148	18168
6	Cr/Yb	[SiO_2_]_adj_/[FeO]_adj_	−481	−3.53	−43.5	18167
7	[MgO]_adj_/Eu	[SiO_2_]_adj_/V	−224	−96	−25.3	398
8	[MgO]_adj_/Hf	[SiO_2_]_adj_/V	−225	−451	−3.61	398
9	[CaO]_adj_/Ta	[SiO_2_]_adj_/V	149	−66	12.7	247
10	[CaO]_adj_/Zr	[SiO_2_]_adj_/V	149	−16840	280	247
11	Ga/Ni	[SiO_2_]_adj_/V	2038	2600	−5518	167
12	Ga/Rb	[SiO_2_]_adj_/V	2037	−8280	1685	167

**Table 8:** Results of the application of significance tests of Fisher *F* and Student *t* to the acid rock data from the Sierra de las Cruces at the strict 99% confidence level (CL) prepared from Excel output of UDASYS [37].

Element	Group A	Group B	*n*_A_	*n*_B_	Df	Sign	*t*_calc	*t*_criteria One-sided	H_0_ One-sided	CL_*t* One-sided	*t*_criteria Two-sided	H_0_ Two-sided	CL_*t* Two-sided
*(a) Major elements *													
[SiO_2_]_adj_	Gr 2	Gr 1	32	11	41.0	−	0.471	2.421	True	<50	2.701	True	<50
[TiO_2_]_adj_	Gr 2	Gr 1	32	11	41.0	−	0.509	2.421	True	<50	2.701	True	<50
[Al_2_O_3_]_adj_	Gr 2	Gr 1	32	11	41.0	−	0.687	2.421	True	72.5	2.701	True	44.9
[Fe_2_O_3_]_adj_	Gr 2	Gr 1	32	11	41.0	+	0.571	2.421	True	<50	2.701	True	<50
[FeO]_adj_	Gr 2	Gr 1	32	11	41.0	+	0.575	2.421	True	<50	2.701	True	<50
[MnO]_adj_	Gr 2	Gr 1	32	11	12.0	+	1.599	2.680	True	93.2	3.053	True	86.4
[MgO]_adj_	Gr 2	Gr 1	32	11	41.0	+	0.612	2.421	True	<50	2.701	True	<50
[CaO]_adj_	Gr 2	Gr 1	32	11	41.0	+	0.602	2.421	True	<50	2.701	True	<50
[Na_2_O]_adj_	Gr 2	Gr 1	32	11	41.0	−	1.877	2.421	True	96.6	2.701	True	93.2
[K_2_O]_adj_	Gr 2	Gr 1	32	11	41.0	+	0.917	2.421	True	81.7	2.701	True	63.3
[P_2_O_5_]_adj_	Gr 2	Gr 1	31	11	40.0	+	0.431	2.423	True	<50	2.705	True	<50
*(b) Trace elements *													
La	Gr 2	Gr 1	32	10	39.1	+	2.661	2.425	False	99.4	2.707	True	98.9
Ce	Gr 2	Gr 1	32	11	41.0	+	1.915	2.421	True	96.9	2.701	True	93.8
Pr	Gr 2	Gr 1	32	10	40.0	+	2.507	2.423	False	99.2	2.705	True	98.4
Nd	Gr 2	Gr 1	31	10	38.9	+	2.117	2.426	True	98.0	2.708	True	95.9
Sm	Gr 2	Gr 1	31	10	37.9	+	1.454	2.429	True	92.3	2.713	True	84.6
Eu	Gr 2	Gr 1	30	10	37.6	+	0.909	2.430	True	81.4	2.713	True	62.8
Gd	Gr 2	Gr 1	31	11	40.0	+	0.096	2.423	True	<50	2.704	True	<50
Tb	Gr 2	Gr 1	31	11	40.0	+	0.144	2.423	True	<50	2.704	True	<50
Dy	Gr 2	Gr 1	31	11	40.0	+	0.331	2.423	True	<50	2.704	True	<50
Ho	Gr 2	Gr 1	31	11	40.0	+	0.503	2.423	True	<50	2.704	True	<50
Er	Gr 2	Gr 1	31	11	40.0	+	0.147	2.423	True	<50	2.704	True	<50
Tm	Gr 2	Gr 1	31	11	40.0	+	0.243	2.423	True	<50	2.704	True	<50
Yb	Gr 2	Gr 1	31	11	40.0	+	0.590	2.423	True	<50	2.704	True	<50
Lu	Gr 2	Gr 1	32	11	41.0	+	0.996	2.421	True	83.7	2.701	True	67.5
Ba	Gr 2	Gr 1	32	11	41.0	+	1.433	2.421	True	92.0	2.701	True	84.0
Be	Gr 2	Gr 1	31	9	38.0	+	1.070	2.429	True	85.5	2.712	True	70.9
Co	Gr 2	Gr 1	32	10	40.0	+	1.330	2.423	True	90.5	2.705	True	80.9
Cr	Gr 2	Gr 1	30	11	39.0	+	0.511	2.423	True	<50	2.708	True	<50
Cs	Gr 2	Gr 1	32	11	41.0	+	1.297	2.421	True	89.9	2.701	True	79.8
Cu	Gr 2	Gr 1	27	11	36.0	−	0.180	2.434	True	<50	2.720	True	<50
Ga	Gr 2	Gr 1	32	11	41.0	+	0.817	2.421	True	78.5	2.701	True	57.0
Hf	Gr 2	Gr 1	32	11	41.0	+	1.305	2.421	True	90.0	2.701	True	80.1
Nb	Gr 2	Gr 1	30	11	39.0	+	1.425	2.426	True	91.9	2.708	True	83.8
Ni	Gr 2	Gr 1	27	9	34.0	+	0.583	2.441	True	<50	2.728	True	<50
Pb	Gr 2	Gr 1	31	11	40.0	+	1.226	2.423	True	88.6	2.704	True	77.3
Rb	Gr 2	Gr 1	32	11	41.0	+	0.735	2.421	True	75.2	2.701	True	50.5
Sb	Gr 2	Gr 1	32	11	41.0	+	0.809	2.421	True	78.2	2.701	True	56.5
Sc	Gr 2	Gr 1	32	11	41.0	+	1.282	2.421	True	89.7	2.701	True	79.3
Sr	Gr 2	Gr 1	32	10	40.0	+	1.528	2.423	True	93.3	2.705	True	86.6
Ta	Gr 2	Gr 1	32	10	40.0	+	1.528	2.423	True	93.3	2.705	True	86.6
Th	Gr 2	Gr 1	32	10	40.0	+	2.216	2.423	True	98.4	2.705	True	96.8
Tl	Gr 2	Gr 1	32	11	41.0	−	0.509	2.421	True	<50	2.701	True	<50
U	Gr 2	Gr 1	32	10	40.0	+	1.954	2.423	True	97.1	2.705	True	94.2
V	Gr 2	Gr 1	32	11	41.0	+	0.533	2.421	True	<50	2.701	True	<50
Y	Gr 2	Gr 1	31	10	39.0	−	0.018	2.426	True	<50	2.708	True	<50
Zr	Gr 2	Gr 1	32	11	41.0	+	0.478	2.421	True	<50	2.701	True	<50
*(c) Geochemical ratios* ^ a^													
LILE4_LREE3	Gr 2	Gr 1	32	11	41.0	−	0.787	2.421	True	77.4	2.701	True	54.8
LILE4_HFSE4	Gr 2	Gr 1	32	11	41.0	+	0.091	2.421	True	<50	2.701	True	<50
Nb_anomaly	Gr 2	Gr 1	31	11	40.0	+	0.421	2.423	True	<50	2.705	True	<50

^a^LILE4_LREE3 = [(K + Rb + Ba + Sr)/4]/[(La + Ce + Nd)/3]; LILE4_HFSE4 = [(K + Rb + Ba + Sr)/4]/[(Ti + P + Nb + Zr)/4]. Nb_anomaly = {Nb/Nb*}_pm_ = [2 ×(Nb_sa_/Nb_pm_)]/[(Ba_sa_/Ba_pm_) + (La_sa_/La_pm_)]; the subscript sa stands for the sample and pm for the primitive mantle; the superscript ∗ refers to the Nb concentration that would result from a smooth pattern for Ba to La on a primitive mantle-normalized multielement diagram [38].
